# Rapid Hemi-Synthesis of Multifunctional Amphiphilic Derivatives Based on Polyphenolic Extractives: Surface Activity, Antioxidant and Antibacterial Properties

**DOI:** 10.3390/molecules30214223

**Published:** 2025-10-29

**Authors:** Maria Celeste Ruiz, Pauline Gérardin, Georges Eid, Jean-Luc Blin, Catherine Humeau-Virot, Christine Gérardin-Charbonnier

**Affiliations:** 1 Laboratoire d’Etudes et de Recherche sur le Matériau Bois (LERMAB), Faculté des Sciences et Technologies, Université de Lorraine, 54506 Vandœuvre-lès-Nancy, France; maria-celeste.ruiz@univ-lorraine.fr (M.C.R.); pauline.gerardin@gmail.com (P.G.); georges.eid@univ-lorraine.fr (G.E.); 2 Laboratoire Lorrain de Chimie Moléculaire, Faculté des Sciences et Technologies, Université de Lorraine, 54506 Vandoeuvre-lès-Nancy, France; jean-luc.blin@univ-lorraine.fr; 3 Laboratoire Réactions et Génie des Procédés (LRGP), ENSAIA, Université de Lorraine, 54506 Vandœuvre-lès-Nancy, France; catherine.humeau@univ-lorraine.fr

**Keywords:** flavonoid, functionalisation, plant chemistry, wood extractives, bioeconomy, renewable resources, hemi-synthesis, polyfunctional compounds, simplified cosmetic formulations

## Abstract

Growing concerns within the petrochemical industry regarding the security and sustainability of supply sources have prompted a search for alternative solutions. In this context, research focused on plant-based chemistry aims to develop molecules that can be transformed into new materials, biotechnological tools or industrial alternatives to fossil compounds. In addition, society and cosmetics companies are seeking to simplify formulations. Accordingly, we provide compounds that combine several functionalities, providing 2-in-1 or even 3-in-1 products. We present here a green hemi-synthetic strategy to obtain original polyfunctional derivatives of catechin by grafting fatty acid or fatty-alanine compounds. These bi-modular and tri-modular compounds exhibit surface activity and radical scavenging activity. They show significant antibacterial activity against *E. coli* as well.

## 1. Introduction

Polyphenols are the main secondary plant metabolites. They are characterized by the presence of at least one aromatic ring with one or more phenolic alcohols, which confers them a great structural and functional diversity [1]. They possess many important biological activities, such as antioxidant, cardioprotective, anticancer, anti-aging, anti-inflammatory and antimicrobial [2,3]. Regarding the antibacterial properties, the position and number of phenolic alcohols in polyphenols is linked to the toxicity they cause in microorganisms [4].

(+)-Catechin is a flavonoid, more specifically a flavan-3-ol, a kind of polyphenol widely found in plants. It shows very good biological activities including antioxidant and antimicrobial. Catechin can inhibit bacterial growth by a variety of mechanisms, including inhibition of nucleic acid synthesis and disruption of cytoplasmic membrane functions [4]. This may result from its interaction with extracellular proteins [5], or from the disruption of cellular proteins [6]. However, the therapeutic potential of catechin and polyphenols, in general, is limited by intrinsic obstacles such as low lipophilicity and low bioavailability [7,8,9].

In order to increase the bioavailability of catechin, chemical or enzymatic modification of its basic structure is a possible approach [10]. Some examples for catechin derivatives are found in the literature: its conjugation with polysaccharides by free radical-mediated reaction [11] or acid condensation reaction [12]; its covalent cross-linking with rice bran protein [13]; the esterification of its phenolic alcohols by acetylation using various benzoyl chlorides [14]; and the esterification of its aliphatic alcohol by enzymatic reaction using different fatty acids [15].

To the best of our knowledge, microwave-assisted esterification of catechin with a fatty acid, or the use of an amino acid such as alanine as a linker between catechin and a fatty acid, has not yet been reported. In both cases, the prior protection of the phenolic alcohols of catechin is required. Different kinds of protection of the phenolic alcohols have been well-studied for a long time [16,17]; however, mainly benzylation is found in the literature for the particular case of catechin. Benzylation reaction involves a number of disadvantages, starting with the use of benzyl chloride or bromide, which are toxic and irritating compounds. Furthermore, these compounds must be used in excess to ensure benzylation of all phenolic alcohols. Even by doing this, the result is a mixture of products that includes di- and tri-benzylated catechin, which means that further purification by column chromatography is necessary to obtain the tetra-benzylated product. The yield of benzylation reaction generally does not exceed 50% [18,19].

Acetylation reaction is a promising candidate to protect phenolic alcohols. Acetylation reaction of catechin and flavonoids, in general, is used to acetylate all the hydroxyl groups in the molecule, phenolic and aliphatic ones. It usually involves the use of acetic anhydride and pyridine [20,21].

In this paper, we present a new strategy for acetylation of the phenolic alcohols of catechin, without acetylation of the aliphatic alcohol, to obtain tetra-acetylated catechin with a yield of 96%. Four different fatty acids or four different alanine–fatty acid couplings are then incorporated by microwave-assisted esterification reaction by acidic catalyst and using either THF or Me-THF as solvent. The bi-modular and tri-modular derivatives have been hemi-synthesized from (+)-catechin on the basis of a green synthesis approach. All of them show a lower water solubility than catechin and all of them can lower the surface tension of water to values between 32 and 49 mN/m. Both of these results could help to increase the bioavailability of catechin. They also exhibit good radical scavenging activity, comparable to that of catechin or other natural polyphenols or synthetic phenolic antioxidants. They show antibacterial activity against *E. coli* as well, comparable to that of catechin, although this property decreases considerably with the longer fatty chain, especially in the tri-modular derivative.

## 2. Results and Discussion

### 2.1. Hemi-Synthesis Reactions and Characterization

The protection of phenolic groups by acetylation reaction is better suited to the requirements of green chemistry when compared to other protections such as benzylation, one of the most commonly used for these kind of compounds [18,19]. Benzyl bromide and benzyl chloride, the key reagents for the benzylation reaction, are highly irritating compounds that can cause skin corrosion and serious eye damage. Moreover, these reagents must be used in excess to ensure benzylation of all phenolic hydroxyl groups. The purification of tetra-benzylated catechin involves a column chromatography, as opposed to the case of tetra-acetylated catechin (**1**), which is performed by recrystallization in water. It should be noted that column chromatography purification is absolutely necessary since reaction produces bi-benzylated and tri-benzylated catechin as by-products.

**Scheme 1:**
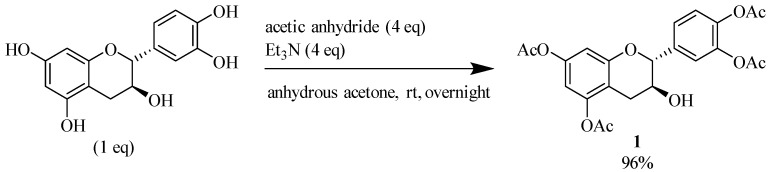
Acetylation reaction of the phenolic hydroxyl groups of (+)-catechin by acetic anhydride and triethylamine (Et_3_N).

So, the first step of the hemi-synthesis strategy, here, is the protection of the phenolic hydroxyl groups of commercially obtained (+)-catechin by acetylation reaction (Scheme 1). To the best of our knowledge, there is only one reference of a tetra-acetylated catechin in the literature. This acetylation takes place using five equivalents of acetyl chloride and five equivalents of triethylamine and gives a tetra-acetylated product with a yield of 35% [21].

With our strategy, compound **1** was obtained with very high yield (96%) after purification by recrystallisation in water.

**Scheme 2:**
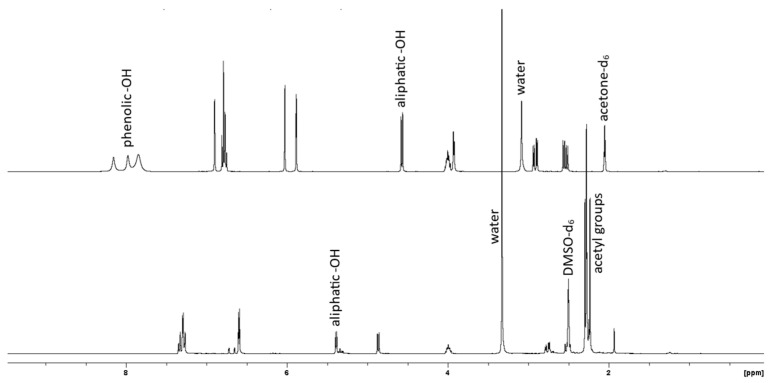
^1^H-NMR spectra in acetone-*d*_6_ of catechin (**top**), and in DMSO-*d*_6_ of compound **1**, catechin with its phenolic hydroxyl groups protected by acetylation reaction (**bottom**); the disappearance of phenolic hydroxyl groups and the persistence of the aliphatic hydroxyl signal are observed.

Acetylation of the phenolic hydroxyl groups of catechin has been verified by ^1^H-NMR by comparison of the spectra of unmodified catechin and compound **1** (Scheme 2). For compound **1**, the presence of the CH_3_ hydrogens of the acetyl groups is observed by the signals at chemical shifts between 2.233 and 2.295 ppm. Moreover, the doublet that integrates for 1 hydrogen at 5.389 ppm corresponds to the signal of the aliphatic hydroxyl group, which is observed for catechin at 4.574 ppm. Based on the obtained data, and the disappearance of the signals corresponding to the phenolic hydroxyls that can be observed at chemical shifts of between 7.858 and 8.163 ppm for catechin, we can conclude that only the acetylation of the phenolic hydroxyl groups has taken place.

**Scheme 3:**
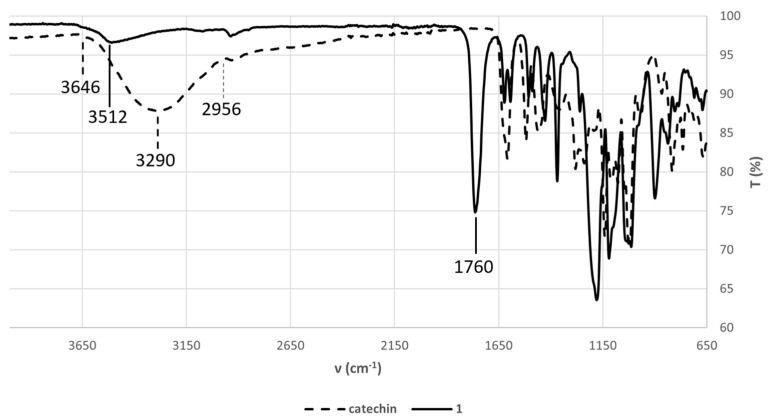
FTIR spectra of catechin (**short dashed line**) and compound **1,** catechin with its phenolic hydroxyl groups protected by acetylation reaction (**solid line**).

Acetylation of the phenolic hydroxyl groups of catechin has also been evidenced by the change in FTIR spectra once the reaction was completed (Scheme 3). The new band observed for compound **1** at 1760 cm^−1^, typical of the carbonyl group of esters (C=O), demonstrates the introduction of new ester bonds resulting from the acetylation reaction. Additionally, the broad band in catechin between 3646 and 2959 cm^−1^ that indicates the presence of alcohol groups (-OH) is reduced to a much narrower band at 3512 cm^−1^ for compound **1**. This may indicate that the amount of hydroxyl groups has been decreased, in this case from five in catechin to only one in compound **1**.

The regioselectivity of this reaction, in which only the phenolic hydroxyl groups are acetylated and not the aliphatic hydroxyl group, can be explained by the use of Et_3_N. It is important to highlight that the equivalents of Et_3_N and acetic anhydride used, as well as the reaction conditions chosen, are essential to ensure this regioselectivity. Aliphatic alcohols have a pKa value of about 16, whereas phenolic ones have a pKa value of about 10. Therefore, the latter can be easier deprotonated by a base such as Et_3_N and then, phenate reacts more rapidly with acetic anhydride.

**Scheme 4:**
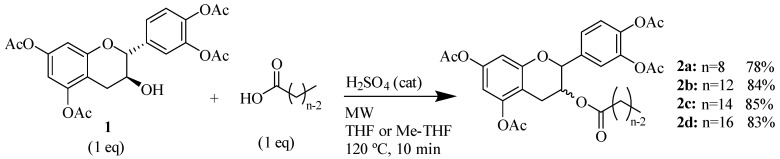
Grafting of a fatty with **1** in microwave-assisted reaction using one drop of H_2_SO_4_ as a catalyst. Octanoic, dodecanoic, tetradecanoic and hexadecanoic acid were used.

Once compound **1** is obtained, it is possible to graft a fatty acid onto the aliphatic alcohol by acid-catalyzed microwave-assisted reaction (Scheme 4). The use of coupling agents is not required.

**Scheme 5:**
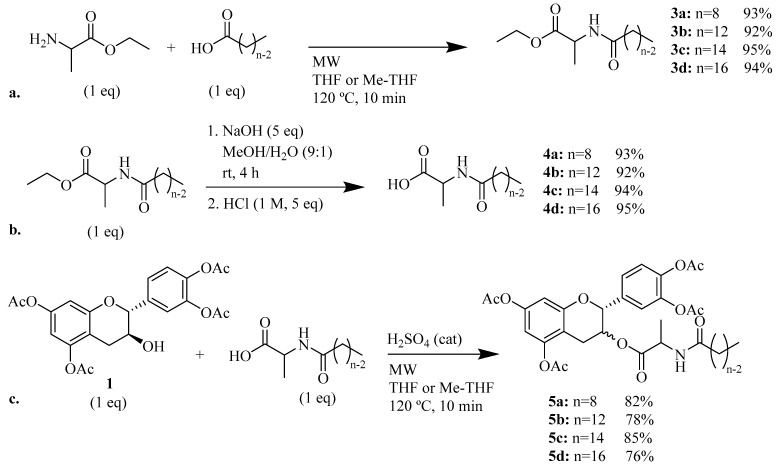
Hemisynthetic strategy for tri-modular compounds catechine–alanine–fatty chain (**a**) grafting an alanine ethyl ester with a fatty acid by microwave-assisted reaction; (**b**) saponification reaction of bi-modular compound. (**c**) Grafting of an alanine–fatty acid coupling with **1** by acid-catalyzed microwave-assisted reaction.

It is also possible to graft a previously obtained amino acid–fatty acid coupling (Scheme 5). For the coupling, an alanine ethyl ester was grafted to a fatty acid by microwave-assisted reaction (Scheme 5a) without any coupling agent. Then, a saponification reaction was performed in order to deprotect the carboxylic acid group from the alanine (Scheme 5b). The coupling obtained was then grafted onto the aliphatic alcohol of compound **1** by acid-catalyzed microwave-assisted reaction (Scheme 5c).

Either for direct grafting or the use of an alanine–fatty acid coupling, four different fatty acids are used: octanoic, dodecanoic, tetradecanoic and hexadecanoic acid. In none of the cases does the length of the carbon chain seem to follow a trend with the reaction yield. Moreover, in all cases, high yields were observed.

The reaction to obtain compounds **2a**–**2d** and **5a**–**5d** is a microwave-assisted esterification reaction that involves the use of H_2_SO_4_ as a catalyst. Compounds **2a**–**2d** and **5a**–**5d** were obtained with high yields.

**Scheme 6:**
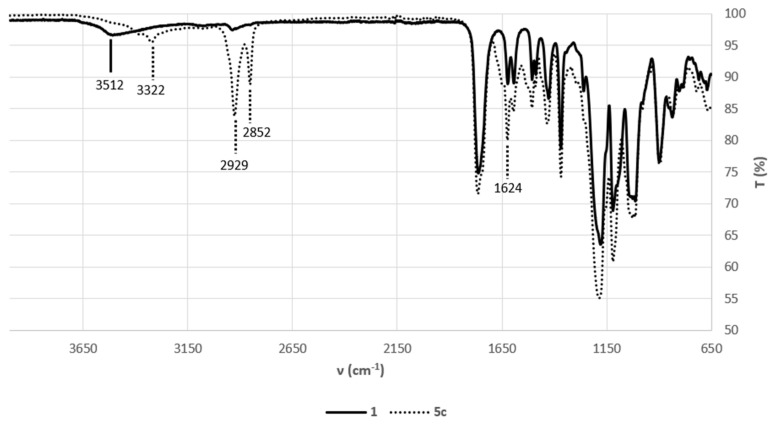
FTIR spectra of compound **1,** catechin with its phenolic hydroxyl groups protected by acetylation reaction (**solid line**) and compound **5c**, a tri-modular acetylated catechin derivative (**dotted line**).

To simplify the analysis, only the FTIR spectra of compounds **1** and **5c**, a tri-modular acetylated catechin derivative, are shown in Scheme 6. The disappearance in the spectra of compound **5c** of the band at 3512 cm^−1^ (aliphatic -OH) allows us to confirm that this group has reacted. Moreover, this compound shows a new band at 1624 cm^−1^ corresponding to the typical signal of a carbonyl (C=O) of an amide group and a band at 3322 cm^−1^ corresponding to the signal of the -NH- group in the amide. Additionally, the bands at 2929 and 2852 cm^−1^ confirm that sp^3^ carbons of type CH, CH_2_ and CH_3_ have been introduced in **5c**, notably a long fatty chain. Finally, the representative band of the carbonyl (C=O) of the newly formed ester group, expected around 1725–1750 cm^−1^, is most likely masked by the broad band of the carbonyls of the acetyl groups at 1760 cm^−1^. This hypothesis may be confirmed in the next and final step of the hemi-synthesis strategy. The same conclusions can be drawn for the rest of the bi- and tri-modular acetylated catechin derivatives, except for the absence of the bands at 1624 cm^−1^ and 3322 cm^−1^ for compounds **2a**–**2d** due to their lack of amide group.

**Scheme 7:**
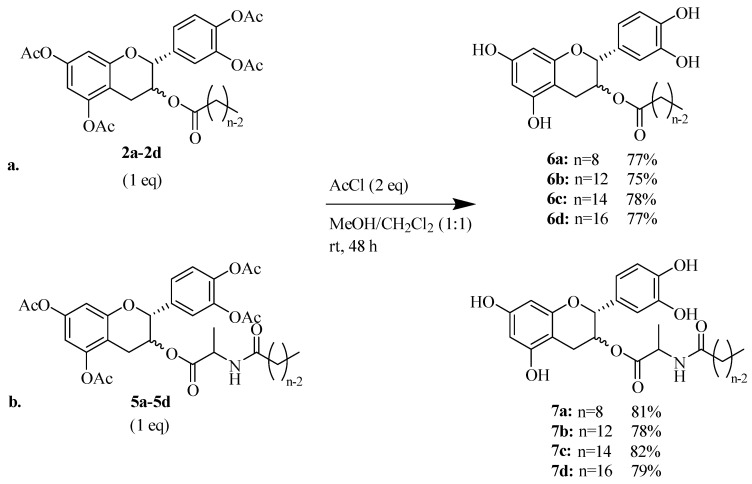
Deacetylation reaction by acetyl chloride (AcCl) to obtain (**a**) the bi-modular catechin derivatives (**6a**–**6d**) and (**b**) the tri-modular catechin derivatives (**7a**–**7d**).

The last step of the hemi-synthesis strategy is to deprotect the acetylated derivatives in order to obtain the final bi-modular (**6a**–**6d**, Scheme 7a) and tri-modular (**7a**–**7d**, Scheme 7b) catechin derivatives. The protocol proposed by Yeom and coworkers [22] was used as a reference, adapting the number of AcCl equivalents to deprotect the four phenolic alcohols (0.5 equivalents per phenol group) and adapting the reaction time. Attempts to use fewer equivalents of AcCl led to partial deacetylation of the catechin derivatives. All final compounds have been obtained with very good yields, and no further purification was needed after the washing with saturated NaHCO_3_ and saturated NaCl once the reaction finished.

**Scheme 8:**
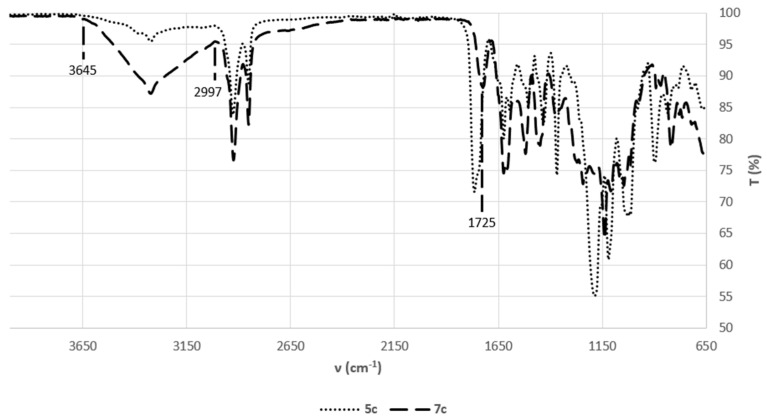
FTIR spectra of compound **5c**, a tri-modular acetylated catechin derivative (**dotted line**) and compound **7c**, a tri-modular catechin derivative (**long dashed line**).

To simplify the analysis, only the FTIR spectra of compounds **5c** and **7c**, a deacetylated tri-modular catechin derivative, are shown in Scheme 8. In compound **7c**, a new broad band between 3645 and 2997 cm^−1^ evidences the presence of alcoholic groups (-OH) in the molecule. The above mentioned and the disappearance of the band at 1760 cm^−1^ that had previously been assigned to the carbonyls (C=O) in the acetyl groups confirm that the deacetylation reaction takes place. Additionally, the band corresponding to the carbonyl (C=O) of the ester is now visible at 1725 cm^−1^ which verifies that for compound **5c** this band was masked by the band of the carbonyls of the acetyl groups. The same conclusions can be drawn for the rest of the final compounds **6a**–**6d** and **7a**,**b**,**d**.

The first step of the deacetylation reaction mechanism may involve the attack of methanol on the carbonyl carbon of the acetyl chloride, leading to the formation of HCl in situ, which can subsequently work as an acid catalyst, and methyl acetate, the by-product of this reaction. The oxygen from the carbonyl of the acetyl group may be protonated by HCl, and then followed by methanol attack on the carbonyl carbon. This may allow the regeneration of both the phenolic alcohol in the final compound and catalyst HCl, as well as the generation of more by-products of methyl acetate. The latter may be eliminated by evaporation in a rotary evaporator. It should be noted that this is a chemoselective reaction, in which only the acetyl groups are attacked, leading to the regeneration of the original phenolic groups. The ester group, less reactive, remains intact.

### 2.2. Water Solubility

Water solubility of anhydrous catechin and the final compounds **6a**–**6d** and **7a**–**7d** are presented in Table 1.

Bi-modular and tri-modular catechin derivatives are soluble in water at all chain lengths. As expected, this solubility decreases as the fatty chain length increases. Bi-modular compounds **6a**–**6d** are more soluble than tri-modular compounds **7a**–**7d** when each fatty chain length is compared. It means that the addition of an amino acid such as alanine increases the lipophilicity of the hemi-synthesis compounds.

Moreover, all bi-modular and tri-modular catechin derivatives are significantly less soluble in water than anhydrous catechin. This increase in lipophilicity in the final compounds could help to increase the bioavailability of catechin.

### 2.3. Surfactant Properties

The surface tension (γ) in water was determined using the Wilhelmy plate method at 25 °C. For the bi-modular and tri-modular catechin derivatives **6a**–**6d** and **7a**–**7d**, it was possible to measure the surface tension at different concentrations in order to obtain the whole curve log C vs. γ. With this curve, parameters such as critical aggregation concentration (CAC) and minimum occupied area per molecule at the air–water interface (σ) were calculated, as well as the surface tension that remains constant from CAC (γ_w_). All the surfactant properties obtained for compounds **6a**–**6d** and **7a**–**7d** are summarized in Table 2.

All compounds **6a**–**6d** and **7a**–**7d** are able to lower the surface tension of water from 72 mN/m to values between 32 and 49 mN/m, which means that they all possess surfactant properties. The introduction of an amino acid such as alanine appears to increase the amphiphilic character of the final compounds and thus to increase their surfactant nature. Compound **7d** shows one of the highest surfactant activities, with both the lowest CAC (2 × 10^−6^ M) and γ_w_ (32 mN/m) values. This means that it requires only a small amount of product to lower the surface tension to around 32 mN/m.

The lower the CAC, the greater the hydrophobic effect, and the more easily the molecules organize themselves together at low concentration. The results show that chain length influences the surface tension decay profile as a function of concentration. The longer the fatty chain, the faster the CAC is reached.

Calculated σ increases as the fatty chain length increases, which explains the fast saturation at the surface. It refers to the smallest possible surface area that a single surfactant molecule occupies when it forms a tightly packed monolayer at the air–water interface. Compounds **6a**–**6d** and **7a**–**7d** present quite low values of σ, which suggests strong intermolecular interactions.

### 2.4. Radical Scavenging Activity

**Scheme 9:**
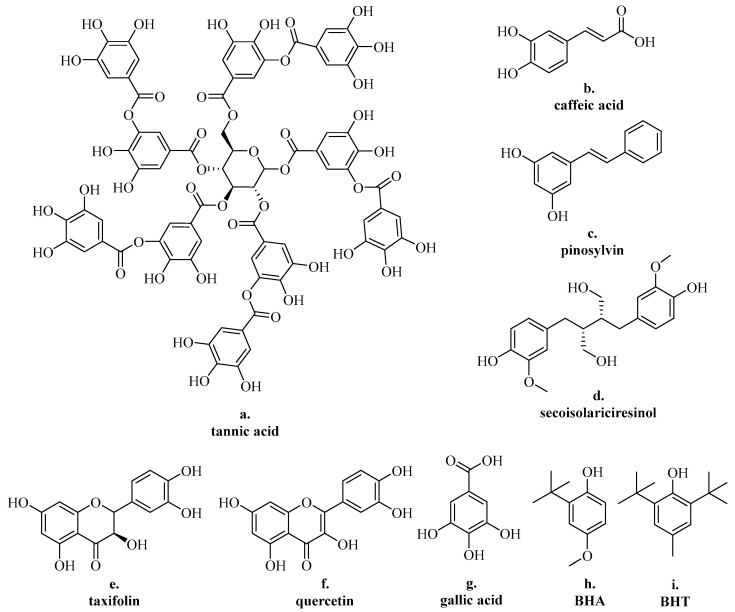
Natural antioxidant polyphenols present in wood (**a**–**g**), and synthetic antioxidant compounds presenting a phenol group (**h**,**i**).

Results for radical scavenging activities by the DPPH method, expressed as IC_50_ values, of catechin, bi-modular (**6a**–**6d**) and tri-modular (**7a**–**7d**) catechin derivatives are presented in Table 3. IC_50_ values found in the literature of other natural antioxidant polyphenols present in wood and two synthetic antioxidant compounds presenting a phenol group (Scheme 9) are also included.

Final products **6a**–**6d** and **7a**–**7c** show comparable radical scavenging activity to that of catechin, with values of IC_50_ between 2.01 and 3.28 μg/mL. The tri-modular catechin derivative with the longest fatty chain **7d** shows the highest IC_50_ value of all the final compounds: 26.73 μg/mL. Nevertheless, this reduction in the antioxidant property is not significant. All final compounds show very good radical scavenging activity, comparable to that of catechin, and to that of other natural polyphenols and synthetic antioxidants presenting a phenol group.

Synthetic antioxidants are widely used due to their purity, low cost and effectiveness even at low concentrations, especially in the pharmaceutical industry and as food additives. However, in the case of long-term use, they may cause some harmful health problems such as carcinogenesis, skin allergies, fatty liver and gastrointestinal distress [24]. The hemi-synthesis of new antioxidants derived from natural products such as polyphenols and amino acids could provide new compounds with good properties similar to the ones shown by the synthetic antioxidants currently used, along with the reduction in undesirable side effects and possible increased biodegradability.

### 2.5. Antibacterial Activity

Antibacterial activity was tested by resazurin reduction test against *E. coli* for anhydrous catechin and all bi-modular and tri-modular catechin derivatives **6a**–**6d** and **7a**–**7d**. These results, expressed as IC_50_ values, are shown in Table 4.

According to the literature, catechins, in general, show good antibacterial properties against different bacteria. These properties are usually studied by dilution and disk diffusion assay [28,29]. The resazurin test is another method that can be used for measuring antibacterial activity [26]. It is a protocol based on cell viability assays [30]. Resazurin is a blue dye that can be irreversibly reduced by the oxidoreductase present in active bacteria. This process leads to the formation of a pink fluorescent substance called resorufin. This reduction allows bacterial metabolic activity to be measured directly by measuring fluorescence. This is used to determine the antibacterial activity of the compounds being tested, using a fluorescence microplate reader. The lower the resorufin production, the lower the measured fluorescence and the higher the antibacterial activity.

We have chosen to study the antibacterial properties against *E. coli*, a Gram-negative bacterium, since it is one of six bio-resistant pathogens that cause more than one million deaths worldwide each year [31]. Moreover, Gram-negative bacteria are characterized by the presence of an extra outer membrane. This is the main reason for their resistance to a wide range of antibiotics. Most of them must cross this outer membrane to reach their targets. Gram-positive bacteria do not have this important layer, which makes Gram-negative bacteria more resistant to antibiotics than Gram-positive ones [32].

Due to their chemical structure, flavonoids can target multiple cellular components in bacteria. Their antibacterial properties stem, in part, from their interactions with cell membranes. Hydrophobic flavonoids are able to penetrate the nonpolar core of the bacterial membrane, while hydrophilic flavonoids may interact with lipid headgroups through hydrogen bonding. They can then either integrate the membrane’s barrier function or induce membrane fusion, which leads to the leakage of intracellular components. Flavonoids can inhibit nucleic acids synthesis as well, mainly by inhibition of several enzymes responsible for this synthesis. Finally, the antibacterial activity of flavonoids includes the prevention or inhibition of biofilm formation [29].

Although catechins generally have lower antibacterial activity than antibiotic drugs, they show great potential for clinical applications. They can enhance the effectiveness of a wide range of antibiotics by working synergistically and restoring antibiotic sensitivity in multidrug-resistant bacterial strains. Additionally, catechins act through multiple mechanisms at the same time, making the development of bacterial resistance less likely. They are also considered biologically safe, with little to no harmful side effects even at high doses, likely because they are a natural part of the human diet [33].

The study of the antibacterial activity of synthetic phenolic antioxidants such as BHA and BHT on various microorganisms has shown that BHA achieves 33% growth of *E. coli* at concentrations above 100 μg/mL. On the other hand, BHT fails to decrease the growth of *E. coli* regardless of the concentration [34].

Chemical modification of catechin, more specifically the introduction of long fatty chains, does not seem to affect the antibacterial power against *E. coli* since IC_50_ values of compounds **6a**–**6c** and **7a**–**7c** are comparable to that of catechin which is 36 mM. They all present IC_50_ values between 23 and 34 mM. However, it seems that the longest fatty chain decreases the antibacterial activity of the derivative significantly, especially when an amino acid such as alanine is introduced. Compound **6d** presents an IC_50_ value of 55 mM and compound **7d** an IC_50_ value of 232 mM, significantly higher than the rest of the derivatives. This decrease in antibacterial activity may be linked to a greater difficulty in these derivatives to penetrate the bacterial cell membrane due to the large fatty chain. The obtained data seem to align with literature findings for saturated fatty acids. Those with chains longer than 14 carbon atoms are found to be moderately efficient antibacterial agents. In cases where these compounds were studied, the MIC values for those with 16 carbon atoms were consistently at least twice as high as those for compounds with 10 carbon atoms. The observed trend may result in a decrease in solubility [35].

The results are promising since the development of both antioxidant and antibacterial new molecules derived from natural products such as polyphenols and amino acids may be of interest to the pharmaceutical, cosmetic and food industries.

## 3. Materials and Methods

### 3.1. General

All reagents other than those synthesized were commercially obtained: (+)-catechin hydrate (CAS: 225937-10-0), l-alanine ethyl ester hydrochloride (CAS: 1115-59-9), acetic anhydride (CAS: 108-24-7), triethylamine (CAS: 121-44-8) and sulfuric acid (CAS: 7664-93-9) were purchased from Sigma-Aldrich (Burlington, VT, USA). Octanoic acid (CAS: 124-07-2), dodecanoic acid (CAS: 143-07-7), tetradecanoic acid (CAS: 544-63-8) and hexadecanoic acid (CAS: 142-62-1) were obtained from Acros Organic (Geel, Belgium). Sodium hydroxide (CAS: 1310-73-2) was obtained from VWR Chemicals (Radnor, PA, United States) and acetyl chloride (CAS: 75-36-5) from Alfa Aesar (Ward Hill, MA, United States).

Microwave-assisted reaction was performed using an Anton Paar Monowave 450 (Anton Paar, Graz, Austria). High-field ^1^H and ^13^C-NMR spectra were obtained using a 400 Bruker Spectrometer (Bruker, Billerica, MA, USA). FTIR spectra were recorded with PERKIN ELMER FTIR Spectrometer (Perkin Elmer, Shelton, WA, USA), by the ATR method. The melting points of the final compounds were determined using a WAGNER & MUNZ HEIZ BANK System Köfler Type VME (Wagner & Münz, Munich, Germany). Elemental analysis was performed for the final compounds.

### 3.2. Hemi-Synthesis Reactions

All reactions were followed by TLC or FTIR.

#### 3.2.1. Procedure for Obtaining Compound **1**

Previously dried, commercially obtained (+)-catechin (4 g, 13.78 mmol, 1 equivalent) was dissolved in anhydrous acetone (80 mL). Acetic anhydride (5.63 g, 55.12 mmol, 4 equivalents) and then Et_3_N (5.58 g, 55.12 mmol, 4 equivalents) were added dropwise. The mixture was stirred at room temperature overnight. Acetone was evaporated under low pressure and CH_2_Cl_2_ (100 mL) was added. The organic phase was washed with saturated NaHCO_3_ (25 mL) until neutral pH of the aqueous phase obtained. Organic phase was dried on anhydrous MgSO_4_ and the solvent was evaporated under low pressure. The residue has been purified by recrystallization in water.


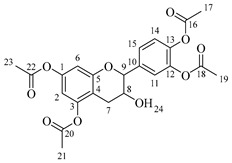


*4-(5,7-Diacetoxy-3-hydroxychroman-2-yl)-1,2-phenylene diacetate* (**1**). C_23_H_22_O_10._ Light orange solid. Yield: 96% (6.06 g). ^1^H-NMR (400 MHz, DMSO-*d*_6_): δ (ppm) = 2.20 (s, 3H, H-17), 2.24 (s, 6H, H-19, H-23), 2.26 (s, 3H, H-21), 2.48 (dd, *J* = 16, 9 Hz, 1H, H-7α), 2.74 (dd, *J* = 16, 5 Hz, 1H, H-7β), 3.92–4.00 (m, 1H, H-8), 4.83 (d, *J* = 8 Hz, 1H, H-9), 5.37 (d, *J* = 5 Hz, H-24), 6.56 (d, *J* = 2 Hz, 1H, H-2), 6.58 (d, *J* = 2 Hz, 1H, H-6), 7.23–7.33 (m, 3H, H-11, H-14, H15). ^13^C-NMR (100 MHz, DMSO-*d*_6_): δ (ppm) = 20.7 (C-17), 20.9 (C-19, C-23), 21.2 (C-21), 28.5 (C-7), 65.5 (C-8), 80.9 (C-9), 107.8 (C-6), 109.1 (C-2), 112.5 (C-4), 122.8 (C-15), 123.8 (C-14), 126.0 (C-11), 138.0 (C-10), 142.1 (C-13), 142.2 (C-12), 149.7 (C-1, C-3), 155.0 (C-5), 168.8 (C-16), 169.1 (C-18, C-22), 169.5 (C-20). IR: ν (cm^−1^) = 1760 (C=O acetyl), 3512 (-OH aliphatic).

#### 3.2.2. Procedure for Obtaining Compounds **2a**–**2d**

Compound **1** (0.5 g, 1.09 mmol, 1 equivalent) and one equivalent of fatty acid were dissolved in 10 mL of anhydrous THF or anhydrous Me-THF. Concentrated H_2_SO_4_ (50 μL, 0.92 mmol) has been added and the mixture has been agitated in a microwave at 120 °C for 10 min using a power of 100 W. Ethyl acetate (100 mL) has been added and washed successively with 25 mL of saturated NaHCO_3_ until neutral pH of the aqueous phase and with 25 mL of saturated NaCl. The organic phase was dried over anhydrous MgSO_4_, filtered and evaporated.


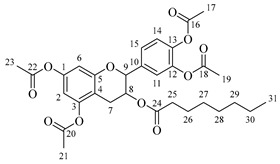


*4-(5,7-Diacetoxy-3-(octanoyloxy)chroman-2-yl)-1,2-phenylene diacetate* (**2a**). C_31_H_36_O_11_. Whitish solid. Yield: 78% (0.497 g). ^1^H-NMR (400 MHz, acetone-*d*_6_): δ (ppm) = 0.88 (t, *J* = 7 Hz, 3H, H-31), 1.26–1.38 (ma, 8H, H-27-30), 1.59 (q, *J* = 7 Hz, 2H, H-26), 2.23 (s, 3H, H-17), 2.27 (s, 6H, H-19, H-23), 2.28 (t, *J* = 7 Hz, 2H, H-25), 2.30 (s, 3H, H-21), 2.57–2.65 (m, 1H, H-7α), 2.88–2.95 (dd, *J* = 16, 5 Hz, 1H, H-7β), 4.06–4.14 (m, 1H, H-8), 4.85 (d, *J* = 8 Hz, 1H, H-9), 6.55 (d, *J* = 2 Hz, 1H, H-2), 6.57 (d, *J* = 2Hz, 1H, H-6), 7.23–7.41 (ma, 3H, H-11, H-14, H-15). ^13^C-NMR (100 MHz, CDCl_3_): δ (ppm) = 14.0 (C-31), 20.6 (C-17), 20.7 (C-19, C-23), 21.0 (C-21), 22.6 (C-30), 24.7 (C-7), 28.2 (C-29), 28.9 (C-28), 29.0 (C-27), 31.6 (C-26), 33.9 (C-25), 67.6 (C-8), 81.2 (C-9), 107.8 (C-2), 108.7 (C-6), 111.7 (C-4), 122.4 (C-15), 123.7 (C-14), 125.6 (C-11), 136.7 (C-10), 149.5 (C-13), 149.7 (C-12), 155.0 (C-1, C-3), 168.4 (C-16), 168.5 (C-18, C-22), 168.6 (C-20), 170.3 (C-24). IR: ν (cm^−1^) = 1764 (C=O acetyl), 1740 (C=O ester).


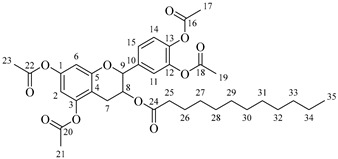


*4-(5,7-Diacetoxy-3-(dodecanoyloxy)chroman-2-yl)-1,2-phenylene diacetate* (**2b**). C_35_H_44_O_11_. Whitish solid. Yield: 84% (0.586 g). ^1^H-NMR (400 MHz, CDCl_3_): δ (ppm) = 0.81 (t, *J* = 7 Hz, 3H, H-35), 1.17–1.31 (ma, 8H, H-27-34), 1.56 (q, *J* = 7 Hz, 2H, H-26), 2.19 (s, 3H, H-17), 2.21 (s, 6H, H-19, H-23), 2.23 (s, 3H, H-21), 2.27 (t, *J* = 7 Hz, 2H, H-25), 2.52–2.59 (m, 1H, H-7α), 2.86–2.94 (dd, *J* = 16, 6 Hz, 1H, H-7β), 3.84–3.92 (m, 1H, H-8), 4.48 (d, *J* = 8 Hz, 1H, H-9), 6.53 (d, *J* = 2 Hz, 1H, H-2), 6.57 (d, *J* = 2Hz, 1H, H-6), 7.09–7.28 (ma, 3H, H-11, H-14, H-15). ^13^C-NMR (100 MHz, CDCl_3_): δ (ppm) = 14.1 (C-35), 20.6 (C-17), 20.7 (C-19, C-23), 21.0 (C-21), 22.6 (C-34), 24.7 (C-7), 28.1 (C-33), 29.0 (C-32), 29.2 (C-31), 29.3 (C-30), 29.4 (C-29), 29.6 (C28-27), 31.9 (C-26), 33.8 (C-25), 67.6 (C-8), 81.1 (C-9), 107.8 (C-2), 108.7 (C-6), 111.7 (C-4), 122.3 (C-15), 123.4 (C-14), 125.5 (C-11), 136.6 (C-10), 142.2 (C-13), 142.3 (C-12), 149.7 (C-1), 155.0 (C-3), 168.3 (C-16), 168.4 (C-18), 168.5 (C-22), 169.0 (C-20), 170.1 (C-24). IR: ν (cm^−1^) = 1763 (C=O acetyl), 1740 (C=O ester).


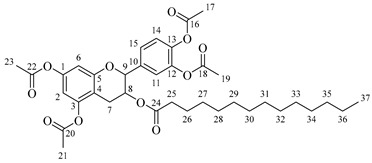


*4-(5,7-Diacetoxy-3-(tetradecanoyloxy)chroman-2-yl)-1,2-phenylene diacetate* (**2c**). C_37_H_48_O_11_. Whitish solid. Yield: 85% (0.619 g). ^1^H-NMR (400 MHz, CDCl_3_): δ (ppm) = 0.80 (t, *J* = 7 Hz, 3H, H-37), 1.16–1.31 (ma, 10H, H-27-36), 1.55 (q, *J* = 7 Hz, 2H, H-26), 2.20 (s, 3H, H-17), 2.22 (s, 6H, H-19, H-23), 2.23 (s, 3H, H-21), 2.28 (t, *J* = 7 Hz, 2H, H-25), 2.52–2.60 (m, 1H, H-7α), 2.86–2.94 (dd, *J* = 16, 6 Hz, 1H, H-7β), 3.85–3.93 (m, 1H, H-8), 4.49 (d, *J* = 8 Hz, 1H, H-9), 6.54 (d, *J* = 2 Hz, 1H, H-2), 6.58 (d, *J* = 2Hz, 1H, H-6), 7.10–7.27 (ma, 3H, H-11, H-14, H-15). ^13^C-NMR (100 MHz, CDCl_3_): δ (ppm) = 14.0 (C-37), 20.7 (C-17), 20.9 (C-19, C-23), 21.0 (C-21), 22.4 (C-36), 24.6 (C-7), 28.2 (C-35), 29.1 (C-34), 29.2 (C-33), 29.3 (C-32), 29.4 (C-31), 29.5 (C-30), 29.6 (C29-27), 31.9 (C-26), 33.7 (C-25), 67.5 (C-8), 81.3 (C-9), 107.9 (C-2), 108.6 (C-6), 111.6 (C-4), 122.4 (C-15), 123.4 (C-14), 125.6 (C-11), 136.7 (C-10), 142.1 (C-13), 142.3 (C-12), 149.6 (C-1), 155.1 (C-3), 168.2 (C-16), 168.4 (C-18), 168.5 (C-22), 169.0 (C-20), 170.3 (C-24). IR: ν (cm^−1^) = 1763 (C=O acetyl), 1741 (C=O ester).


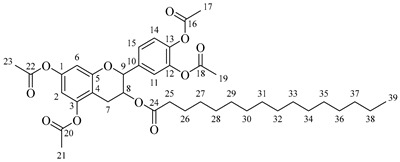


*4-(5,7-Diacetoxy-3-(hexadecanoyloxy)chroman-2-yl)-1,2-phenylene diacetate* (**2d**). C_39_H_52_O_11_. Whitish solid. Yield: 83% (0.630 g). ^1^H-NMR (400 MHz, CDCl_3_): δ (ppm) = 0.81 (t, *J* = 7 Hz, 3H, H-39), 1.12–1.31 (ma, 2H, H-27-38), 1.56 (q, *J* = 7 Hz, 2H, H-26), 2.21 (s, 3H, H-17), 2.22 (s, 6H, H-19, H-23), 2.24 (s, 3H, H-21), 2.28 (t, *J* = 7 Hz, 2H, H-25), 2.51–2.64 (m, 1H, H-7α), 2.89–2.96 (dd, *J* = 16, 5 Hz, 1H, H-7β), 3.86–3.94 (m, 1H, H-8), 4.65 (d, *J* = 8 Hz, 1H, H-9), 6.48 (d, *J* = 2 Hz, 1H, H-2), 6.53 (d, *J* = 2Hz, 1H, H-6), 7.09–7.30 (ma, 3H, H-11, H-14, H-15). ^13^C-NMR (100 MHz, CDCl_3_): δ (ppm) = 14.1 (C-39), 20.6 (C-17), 20.7 (C-19, C-23), 21.0 (C-21), 22.7 (C-38), 24.7 (C-7), 29.0 (C-37), 29.2 (C-36), 29.3 (C-35), 29.4 (C-34), 29.5 (C-33), 29.6 (C-32), 29.7 (C27-31), 31.9 (C-26), 33.7 (C-25), 67.7 (C-8), 81.1 (C-9), 107.8 (C-2), 108.8 (C-6), 111.7 (C-4), 122.4 (C-15), 123.7 (C-14), 125.6 (C-11), 136.7 (C-10), 149.5 (C-13), 149.7 (C-12), 155.0 (C-1, C-3), 168.4 (C-16), 168.5 (C-18, C-22), 168.6 (C-20), 178.0 (C-24). IR: ν (cm^−1^) = 1764 (C=O acetyl), 1741 (C=O ester).

#### 3.2.3. Procedure for Obtaining Compounds **3a**–**3d**

l-alanine ethyl ester (1 g, 6.51 mmol, 1 equivalent) and one equivalent of fatty acid have been dissolved in 15 mL of anhydrous THF or anhydrous Me-THF. The mixture has been stirred in a microwave at 120 °C for 10 min using a power of 100 W. A white solid has then been filtrated and washed with 20 mL of anhydrous THF or MeTHF.


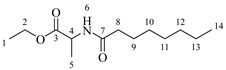


*Ethyl octanoylalaninate* (**3a**). C_13_H_25_NO_3_. White solid. Yield: 93% (1.472 g). ^1^H-NMR (400 MHz, CDCl_3_): δ (ppm) = 0.82 (t, *J* = 7 Hz, 3H, H-14), 1.11–1.29 (ma, 11H, H-10-13, H-1), 1.32 (d, *J* = 7 Hz, 3H, H-5), 1.48–1.63 (ma, 2H, H-9), 2.13 (t, *J* = 7 Hz, 2H, H-8), 4.13 (q, *J* = 7 Hz, 2H, H-2), 4.51 (m, *J* = 7 Hz, 1H, H-4), 6.03 (s, 1H, H-6). ^13^C-NMR (100 MHz, DMSO-*d*_6_): δ (ppm) = 14.1 (C-1, C-14), 17.3 (C-5), 22.7 (C-13), 25.6 (C-9), 28.6 (C-10, C-11), 31.8 (C-12), 36.5 (C-8), 52.1 (C-4), 61.3 (C-2), 171.5 (C-3), 173.9 (C-7). IR: ν (cm^−1^) = 1649 (C=O amide), 1738 (C=O ester), 3317 (-NH- amide).


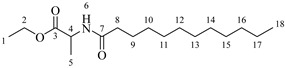


*Ethyl dodecanoylalaninate* (**3b**). C_17_H_33_NO_3_. White solid. Yield: 92% (1.792 g). ^1^H-NMR (400 MHz, CDCl_3_): δ (ppm) = 0.74 (t, *J* = 7 Hz, 3H, H-18), 1.05–1.17 (ma, 19H, H-10-17, H-1), 1.19 (d, *J* = 7 Hz, 3H, H-5), 1.38–1.51 (ma, 2H, H-9), 2.05 (t, *J* = 7 Hz, 2H, H-8), 3.98 (q, *J* = 7 Hz, 2H, H-2), 4.26 (m, *J* = 7 Hz, 1H, H-4), 7.13 (s, 1H, H-6). ^13^C-NMR (100 MHz, DMSO-*d*_6_): δ (ppm) = 14.3 (C-1, C-14), 17.5 (C-5), 23.1 (C-17), 25.2 (C-9), 28.6 (C-10), 28.9 (C-11), 29.3 (C-15), 29.6 (C-12-14), 31.9 (C-16), 36.6 (C-8), 52.0 (C-4), 61.3 (C-2), 172.0 (C-3), 173.9 (C-7). IR: ν (cm^−1^) = 1647 (C=O amide), 1738 (C=O ester), 3315 (-NH- amide).


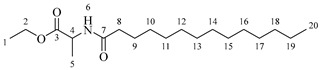


*Ethyl tetradecanoylalaninate* (**3c**). C_19_H_37_NO_3_. White solid. Yield: 95% (2.024 g). ^1^H-NMR (400 MHz, CDCl_3_): δ (ppm) = 0.81 (t, *J* = 7 Hz, 3H, H-20), 1.12–1.29 (ma, 23H, H-10-19, H-1), 1.32 (d, *J* = 7 Hz, 3H, H-5), 1.49–1.64 (ma, 2H, H-9), 2.13 (t, *J* = 7 Hz, 2H, H-8), 4.13 (q, *J* = 7 Hz, 2H, H-2), 4.51 (m, *J* = 7 Hz, 1H, H-4), 5.96 (s, 1H, H-6). ^13^C-NMR (100 MHz, DMSO-*d*_6_): δ (ppm) = 14.0 (C-1, C-20), 17.1 (C-5), 22.5 (C-19), 25.9 (C-9), 28.6 (C-10), 28.9 (C-11), 29.5 (C-17), 29.9 (C-12-16), 32.5 (C-18), 37.5 (C-8), 52.2 (C-4), 62.7 (C-2), 170.2 (C-3), 174.2 (C-7). IR: ν (cm^−1^) = 1648 (C=O amide), 1738 (C=O ester), 3319 (-NH- amide).


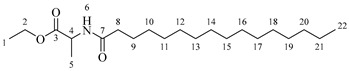


*Ethyl hexadecanoylalaninate* (**3d**). C_21_H_41_NO_3_. White solid. Yield: 94% (2.174 g). ^1^H-NMR (400 MHz, CDCl_3_): δ (ppm) = 0.83 (t, *J* = 7 Hz, 3H, H-22), 1.10–1.34 (ma, 27H, H-10-21, H-1), 1.35 (d, *J* = 7 Hz, 3H, H-5), 1.35–1.49 (ma, 2H, H-9), 2.09 (t, *J* = 7 Hz, 2H, H-8), 4.25 (q, *J* = 7 Hz, 2H, H-2), 4.43 (m, *J* = 7 Hz, 1H, H-4), 6.54 (s, 1H, H-6). ^13^C-NMR (100 MHz, DMSO-*d*_6_): δ (ppm) = 13.9 (C-1, C-22), 17.5 (C-5), 23.1 (C-21), 25.2 (C-9), 28.8 (C-10), 29.3 (C-11), 29.5 (C-19), 29.8 (C-10-18), 31.5 (C-20), 36.7 (C-8), 53.6 (C-4), 61.7 (C-2), 171.2 (C-3), 173.7 (C-7). IR: ν (cm^−1^) = 1644 (C=O amide), 1735 (C=O ester), 3315 (-NH- amide).

#### 3.2.4. Procedure for Obtaining Compounds **4a**–**4d**

Alanine–fatty acid coupling (1 g) has been dissolved in a solution of five equivalents of NaOH in 90 mL of a mixture of MeOH/H_2_O (9:1). After two hours of magnetic agitation at room temperature, a white precipitate has been formed. The round bottom flask has been placed in an ice bath and five equivalents of HCl 1 M solution has been added until the white precipitate has formed again. The methanol has been evaporated and the rest of the solution has been lyophilized to obtain the saponification product.


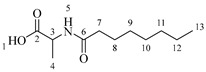


*Octanoylalanine* (**4a**). C_11_H_21_NO_3_. Yellowish liquid. Yield: 93% (0.822 g). ^1^H-NMR (400 MHz, CDCl_3_): δ (ppm) = 0.80 (t, *J* = 7 Hz, 3H, H-13), 1.11–1.33 (ma, 8H, H-9-12), 1.39 (d, *J* = 7 Hz, 3H, H-4), 1.51–1.62 (ma, 2H, H-8), 2.17 (t, *J* = 7 Hz, 2H, H-7), 4.51 (m, *J* = 7 Hz, 1H, H-3), 6.01 (s, 1H, H-5). ^13^C-NMR (100 MHz, DMSO-*d*_6_): δ (ppm) = 14.1 (C-13), 17.0 (C-4), 22.7 (C-12), 25.6 (C-8), 28.6 (C-9-10), 31.9 (C-11), 36.5 (C-7), 51.3 (C-3), 173.9 (C-6), 174.7 (C-2). IR: ν (cm^−1^) 1644 (C=O amide), 1703 (C=O acid), 3227 (-NH- amide), 3308 (-OH acid).


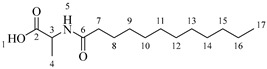


*Dodecanoylalanine* (**4b**). C_15_H_29_NO_3_. White solid. Yield: 92% (0.833 g). ^1^H-NMR (400, MHz, CDCl_3_): δ (ppm) = 0.80 (t, *J* = 7 Hz, 3H, H-17), 1.12–1.33 (ma, 16H, H-9-16), 1.40 (d, *J* = 7 Hz, 3H, H-4), 1.51–1.62 (ma, 2H, H-8), 2.16 (t, *J* = 7 Hz, 2H, H-7), 4.50 (m, *J* = 7 Hz, 1H, H-3), 6.25 (s, 1H, H-5). ^13^C-NMR (100 MHz, DMSO-*d*_6_): δ (ppm) = 14.1 (C-17), 17.0 (C-4), 22.7 (C-16), 25.6 (C-8), 28.6 (C-9), 28.9 (C-10), 29.6 (C-11-13), 29.3 (C-14), 31.9 (C-15), 36.5 (C-7), 51.3 (C-3), 173.9 (C-6), 174.7 (C-2). IR: ν (cm^−1^) = 1643 (C=O amide), 1702 (C=O acid), 3229 (-NH- amide), 3311 (-OH acid).


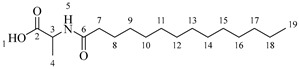


*Tetradecanoylalanine* (**4c**). C_17_H_33_NO_3_. White solid. Yield: 94% (0.861 g). ^1^H-NMR (400 MHz, CDCl_3_): δ (ppm) = 0.82 (t, *J* = 7 Hz, 3H, H-19), 1.1–1.33 (ma, 20H, H-9-18), 1.39 (d, *J* = 7 Hz, 3H, H-4), 1.52–1.62 (ma, 2H, H-8), 2.17 (t, *J* = 7 Hz, 2H, H-7), 4.49 (m, *J* = 7 Hz, 1H, H-3), 6.12 (s, 1H, H-5). ^13^C-NMR (100 MHz, DMSO-*d*_6_): δ (ppm) = 14 (C-19), 17.2 (C-4), 22.5 (C-18), 25.6 (C-18), 28.6 (C-9), 28.9 (C-10), 29.6 (C-11-15), 29.3 (C-16), 31.9 (C-17), 36.6 (C-7), 51.3 (C-3), 174.0 (C-6), 174.7 (C-2). IR: ν (cm^−1^) = 1644 (C=O amide), 1705 (C=O acid), 3227 (-NH- amide), 3315 (-OH acid).


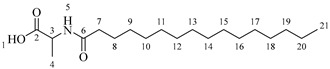


*Hexadecanoylalanine* (**4d**). C_19_H_37_NO_3_. White solid. Yield: 95% (0.874 g). ^1^H-NMR (400 MHz, CDCl_3_): δ (ppm) = 0.79 (t, *J* = 7 Hz, 3H, H-21), 1.12–1.31 (ma, 24H, H-9-20), 1.41 (d, *J* = 7 Hz, 3H, H-4), 1.52–1.62 (ma, 2H, H-8), 2.15 (t, *J* = 7 Hz, 2H, H-7), 4.51 (m, *J* = 7 Hz, 1H, H-3), 6.07 (s, 1H, H-5). ^13^C-NMR (100 MHz, DMSO-*d*_6_): δ (ppm) = 14.2 (C-21), 17.1 (C-4), 22.7 (C-20), 25.6 (C-8), 28.6 (C-9), 28.9 (C-10), 29.6 (C-11-17), 29.4 (C-18), 32.0 (C-19), 36.2 (C-7), 51.4 (C-3), 174.0 (C-6), 174.6 (C-2). IR: ν (cm^−1^) = 1644 (C=O amide), 1703 (C=O acid), 3222 (-NH- amide), 3317 (-OH acid)

#### 3.2.5. Procedure for Obtaining Compounds **5a**–**5d**

Compound **1** (0.5 g, 1.09 mmol, 1 equivalent) and one equivalent of alanine–fatty acid coupling have been dissolved in 10 mL of anhydrous THF or anhydrous Me-THF. Concentrated H_2_SO_4_ (50 μL, 0.92 mmol) has been added and the mixture was stirred in a microwave at 120 °C for 10 min using a power of 100 W. An amount of 100 mL of ethyl acetate has been added and the organic phase has been washed successively with 25 mL of saturated NaHCO_3_ (several times if necessary until neutral pH of the aqueous phase) and with 25 mL of saturated NaCl. The organic phase has been dried over anhydrous MgSO_4_ and the solvent has been evaporated under low pressure.


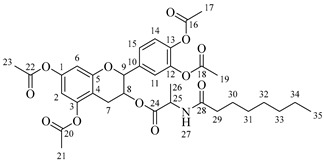


*4-(5,7-Diacetoxy-3-((octanoylalanyl)oxy)chroman-2-yl)-1,2-phenylene diacetate* (**5a**). C_34_H_41_NO_12_. Whitish solid. Yield: 82% (0.585 g). ^1^H-NMR (400 MHz, CDCl_3_): δ (ppm) = 0.81 (t, *J* = 7 Hz, 3H, H-35), 1.18–1.28 (ma, 8H, H-31-34), 1.36 (d, *J* = 7 Hz, 3H, H-26), 1.55 (q, *J* = 7 Hz, 2H, H-30), 2.20 (s, 3H, H-17), 2.21 (s, 6H, H-19, H-23), 2.23 (s, 3H, H-21), 2.26 (t, *J* = 7 Hz, 2H, H-29), 2.53–2.64 (m, 1H, H-7α), 2.87–2.95 (dd, *J* = 16, 6 Hz, 1H, H-7β), 3.85–3.94 (m, 1H, H-8), 4.28 (q, *J* = 7 Hz, 1H, H-25), 4.66 (d, *J* = 8 Hz, 1H, H-9), 6.49 (d, *J* = 2 Hz, 1H, H-2), 6.54 (d, *J* = 2 Hz, 1H, H-6), 6.60 (d, *J* = 6 Hz, 1H, H-27), 7.10–7.29 (ma, 3H, H-11, H-14, H-15). ^13^C-NMR (100 MHz, CDCl_3_): δ (ppm) = 14.0 (C-35), 17.8 (C-26), 20.6 (C-17), 20.7 (C-19, C-23), 21.0 (C-21), 22.7 (C-34), 25.5 (C-7), 28.1 (C-33), 29.1 (C-32), 29.2 (C-31), 31.9 (C-30), 36.3 (C-29), 36.8 (C-25), 67.4 (C-8), 81.1 (C-9), 107.7 (C-2), 108.7 (C-6), 111.6 (C-4), 122.3 (C-15), 123.7 (C-14), 125.5 (C-11), 136.5 (C-10), 142.2 (C-12), 142.3 (C-13), 149.7 (C-1), 155.0 (C-3), 168.0 (C-16), 168.2 (C-18-22), 168.5 (C-20), 170.1 (C-24), 175.0 (C-28). IR: ν (cm^−1^) = 3315 (N-H amide), 1763 (C=O acetyl), 1743 (C=O ester), 1642 (C=O amide).


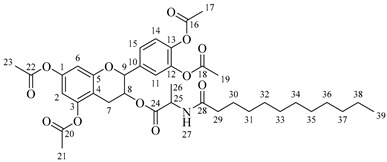


*4-(5,7-Diacetoxy-3-((dodecanoylalanyl)oxy)chroman-2-yl)-1,2-phenylene diacetate* (**5b**). C_38_H_49_NO_12_. Whitish solid. Yield: 78% (0.544 g). ^1^H-NMR (400 MHz, CDCl_3_): δ (ppm) = 0.81 (t, *J* = 7 Hz, 3H, H-39), 1.12–1.29 (ma, 16H, H-31-38), 1.38 (d, *J* = 7 Hz, 3H, H-26), 1.56 (q, *J* = 7 Hz, 2H, H-30), 2.17 (t, *J* = 7 Hz, 2H, H-29), 2.20 (s, 3H, H-17), 2.21 (s, 6H, H-19, H-23), 2.23 (s, 3H, H-21), 2.51–2.63 (m, 1H, H-7α), 2.87–2.95 (dd, *J* = 16, 6 Hz, 1H, H-7β), 3.85–3.94 (m, 1H, H-8), 4.49 (q, *J* = 7 Hz, 1H, H-25), 4.66 (d, *J* = 8 Hz, 1H, H-9), 6.01 (d, *J* = 6 Hz, 1H, H-27), 6.48 (d, *J* = 2 Hz, 1H, H-2), 6.53 (d, *J* = 2 Hz, 1H, H-6), 7.07–7.28 (ma, 3H, H-11, H-14, H-15). ^13^C-NMR (100 MHz, CDCl_3_): δ (ppm) = 14.1 (C-39), 17.8 (C-26), 20.6 (C-17), 20.7 (C-19, C-23), 21.0 (C-21), 22.6 (C-38), 25.5 (C-7), 28.1 (C-37), 29.1 (C-36), 29.2 (C-35), 29.3 (C-34), 29.4, (C-33), 29.6 (C-32-31), 31.9 (C-30), 36.3 (C-29), 48.3 (C-25), 67.6 (C-8), 81.1 (C-9), 107.8 (C-2), 108.7 (C-6), 111.6 (C-4), 122.3 (C-15), 123.7 (C-14), 125.5 (C-11), 136.5 (C-10), 142.3 (C-12-13), 149.5 (C-1), 155.0 (C-3), 168.3 (C-16), 168.4 (C-18-22), 168.6 (C-20), 174.2 (C-24), 175.0 (C-28). IR: ν (cm^−1^) = 3315 (N-H amide), 1766 (C=O acetyl), 1740 (C=O ester), 1642 (C=O amide).


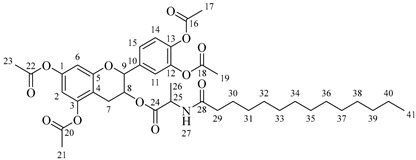


*4-(5,7-Diacetoxy-3-((tetradecanoylalanyl)oxy)chroman-2-yl)-1,2-phenylene diacetate* (**5c**). C_40_H_53_NO_12_. Whitish solid. Yield: 85% (0.619 g). ^1^H-NMR (400 MHz, CDCl_3_): δ (ppm) = 0.82 (t, *J* = 7 Hz, 3H, H-41), 1.14–1.29 (ma, 20H, H-31-40), 1.40 (d, *J* = 7 Hz, 3H, H-26), 1.57 (q, *J* = 7 Hz, 2H, H-30), 2.18 (t, *J* = 7 Hz, 2H, H-29), 2.20 (s, 3H, H-17), 2.22 (s, 6H, H-19, H-23), 2.24 (s, 3H, H-21), 2.54–2.63 (m, 1H, H-7α), 2.88–2.96 (dd, *J* = 16, 6 Hz, 1H, H-7β), 3.88–3.95 (m, 1H, H-8), 4.50 (q, *J* = 7 Hz, 1H, H-25), 4.68 (d, *J* = 8 Hz, 1H, H-9), 5.99 (d, *J* = 7 Hz, 1H, H-27), 6.49 (d, *J* = 2 Hz, 1H, H-2), 6.54 (d, *J* = 2 Hz, 1H, H-6), 7.08–7.30 (ma, 3H, H-11, H-14, H-15). ^13^C-NMR (100 MHz, CDCl_3_): δ (ppm) = 14.1 (C-41), 17.8 (C-26), 20.6 (C-17), 20.7 (C-19, C-23), 21.0 (C-21), 22.7 (C-40), 25.5 (C-7), 28.1 (C-39), 29.1 (C-38), 29.2 (C-37), 29.3 (C-36), 29.4 (C-35), 29.5 (C-34), 29.6 (C-33), 29.7 (C-32-31), 31.9 (C-30), 36.4 (C-29), 48.3 (C-25), 67.6 (C-8), 81.1 (C-9), 107.8 (C-2), 108.8 (C-6), 111.6 (C-4), 122.3 (C-15), 123.7 (C-14), 125.4 (C-11), 136.5 (C-10), 142.2 (C-12), 142.3 (C-13), 149.5 (C-1), 155.0 (C-3), 168.2 (C-16), 168.3 (C-18-22), 168.5 (C-20), 174.2 (C-24), 175.0 (C-28). IR: ν (cm^−1^) = 3312 (N-H amide), 1766 (C=O acetyl), 1741 (C=O ester), 1645 (C=O amide).


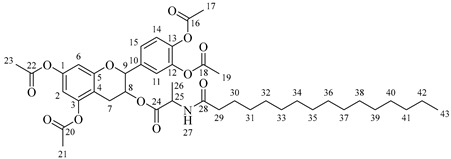


*4-(5,7-Diacetoxy-3-((hexadecanoylalanyl)oxy)chroman-2-yl)-1,2-phenylene diacetate* (**5d**). C_42_H_57_NO_12_. Whitish solid. Yield: 76% (0.577 g). ^1^H-NMR (400 MHz, CDCl3): δ (ppm) = 0.82 (t, *J* = 7 Hz, 3H, H-43), 1.13–1.28 (ma, 24H, H-31-42), 1.38 (d, *J* = 7 Hz, 3H, H-26), 1.56 (q, *J* = 7 Hz, 2H, H-30), 2.16 (t, *J* = 7 Hz, 2H, H-29), 2.20 (s, 3H, H-17), 2.21 (s, 6H, H-19, H-23), 2.24 (s, 3H, H-21), 2.52–2.60 (m, 1H, H-7α), 2.87–2.94 (dd, *J* = 16, 6 Hz, 1H, H-7β), 3.86–3.93 (m, 1H, H-8), 4.50 (q, *J* = 7 Hz, 1H, H-25), 4.66 (d, *J* = 8 Hz, 1H, H-9), 6.02 (d, *J* = 7 Hz, 1H, H-27), 6.48 (d, *J* = 2 Hz, 1H, H-2), 6.53 (d, *J* = 2 Hz, 1H, H-6), 7.13–7.28 (ma, 3H, H-11, H-14, H-15). ^13^C-NMR (100 MHz, CDCl3): δ (ppm) = 14.1 (C-43), 17.8 (C-26), 20.6 (C-17), 20.7 (C-19, C-23), 21.0 (C-21), 22.6 (C-42), 25.5 (C-7), 28.1 (C-41), 29.1 (C-40), 29.2 (C-39), 29.3 (C-38), 29.4 (C-37), 29.5 (C-36), 29.6 (C-35-34), 29.7 (C33-32), 31.9 (C-31), 33.7 (C-30), 36.4 (C-29), 48.2 (C-25), 67.5 (C-8), 81.1 (C-9), 107.7 (C-2), 108.7 (C-6), 111.6 (C-4), 122.3 (C-15), 123.7 (C-14), 125.5 (C-11), 136.5 (C-10), 142.3 (C-12-13), 149.5 (C-1), 155.0 (C-3), 168.3 (C-16), 168.5 (C-18-22), 168.9 (C-20), 174.0 (C-24), 175.1 (C-28). IR: ν (cm^−1^) = 3313 (N-H amide), 1764 (C=O acetyl), 1740 (C=O ester), 1645 (C=O amide).

#### 3.2.6. Procedure for Obtaining Compounds **6a**–**6d** and **7a**–**7d**

Acetylated compound (0.65 mmol, 1 equivalent) has been solubilized in 100 mL of a mixture of MeOH/CH_2_Cl_2_ (1:1). Acetyl chloride (0.102 g, 1.30 mmol, 2 equivalents) has been added dropwise. The mixture has been stirred for 48 h at room temperature. The reaction has been followed by FTIR by evaluation of the disappearance of the band at 1760 cm^−1^. Once there was no longer any starting product, the solvents evaporated. Water was added to precipitate the final product. The purification has been performed by recrystallization in water.


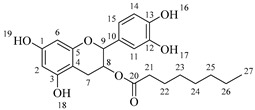


*2-(3,4-Dihydroxyphenyl)-5,7-dihydroxychroman-3-yl octanoate* (**6a**) [36]. Whitish solid. Yield: 77% (0.208 g). m.p. 105 °C. Anal. Calculated for C_23_H_28_O_7_: C, 66.33; H, 6.78; Found: C, 66.78; H, 6.60. ^1^H-NMR (400 MHz, DMSO-*d*_6_): δ (ppm) = 0.84 (t, *J* = 7 Hz, 3H, H-27), 1.20–1.28 (ma, 8H, H-23-26), 1.49 (q, *J* = 7 Hz, 2H, H-22), 2.27 (t, *J* = 7 Hz, 2H, H-21), 2.30–2.38 (m, 1H, H-7α), 2.60–2.68 (dd, *J* = 16, 5 Hz, 1H, H-7β), 3.76–3.86 (m, 1H, H-8), 4.89 (d, *J* = 5 Hz, 1H, H-9), 5.68 (d, *J* = 2 Hz, 1H, H-2), 5.87 (d, *J* = 2 Hz, 1H, H-6), 6.51–6.73 (ma, 3H, H-11, H-14, H-15), 8.84 (s, 1H, H-16), 8.87 (s, 1H, H-17), 8.98 (s, 1H, H-19), 9.22 (s, 1H, H-18). ^13^C-NMR (100 MHz, MeOD): δ (ppm) = 14.4 (C-27), 23.6 (C-26), 26.0 (C-7), 28.5 (C-25), 30.0 (C-24), 30.1 (C-23), 32.8 (C-22), 34.8 (C-21), 68.7 (C-8), 82.8 (C-9), 95.4 (C-2), 96.2 (C-6), 100.7 (C-4), 115.2 (C-15), 116.6 (C-14), 120.0 (C-11), 132.1 (C-10), 145.1 (C-13), 145.2 (C-12), 156.6 (C-1), 157.5 (C-3), 157.7 (C-5), 174.8 (C-20). IR: ν (cm^−1^) = 3000–3676 (-OH), 1742 (C=O ester).


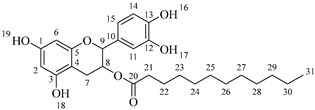


*2-(3,4-Dihydroxyphenyl)-5,7-dihydroxychroman-3-yl dodecanoate* (**6b**) [36]. Whitish solid. Yield: 75% (0.230 g). m.p. 120 °C. Anal. Calculated for C_27_H_36_O_7_: C, 68.62; H, 7.68; Found: C, 68.65; H, 7.48. ^1^H-NMR (400 MHz, DMSO-*d*_6_): δ (ppm) = 0.84 (t, *J* = 7 Hz, 3H, H-31), 1.19–1.29 (ma, 16H, H-23-30), 1.49 (q, *J* = 7 Hz, 2H, H-22), 2.26 (t, *J* = 7 Hz, 2H, H-21), 2.31–2.39 (m, 1H, H-7α), 2.60–2.68 (dd, *J* = 16, 5 Hz, 1H, H-7β), 3.76–3.86 (m, 1H, H-8), 4.89 (d, *J* = 5 Hz, 1H, H-9), 5.68 (d, *J* = 2 Hz, 1H, H-2), 5.87 (d, *J* = 2 Hz, 1H, H-6), 6.51–6.73 (ma, 3H, H-11, H-14, H-15), 8.88 (s, 1H, H-16), 8.98 (s, 1H, H-17), 9.03 (s, 1H, H-19), 9.22 (s, 1H, H-18). ^13^C-NMR (100 MHz, MeOD): δ (ppm) = 14.4 (C-31), 23.7 (C-30), 26.0 (C-7), 28.5 (C-29), 30.1 (C-28), 30.3 (C-27), 30.4 (C-26), 30.5 (C-25), 30.7 (C-24), 30.8 (C-23), 33.0 (C-22), 34.8 (C-21), 68.8 (C-8), 82.8 (C-9), 95.5 (C-2), 96.3 (C-6), 100.8 (C-4), 115.3 (C-15), 116.2 (C-14), 120.0 (C-11), 132.2 (C-10), 144.3 (C-13), 146.4 (C-12), 156.9 (C-1), 158.0 (C-3), 158.5 (C-5), 174.5 (C-20). IR: ν (cm^−1^) = 2994–3700 (-OH), 1742 (C=O ester).


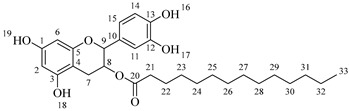


*2-(3,4-Dihydroxyphenyl)-5,7-dihydroxychroman-3-yl tetradecanoate* (**6c**) [36]. Whitish solid. Yield: 78% (0.254 g). m.p. 130 °C. Anal. Calculated for C_29_H_40_O_7_: C, 69.58; H, 8.05; Found: C, 69.52; H, 8.10. ^1^H-NMR (400 MHz, DMSO-*d*_6_): δ (ppm) = 0.84 (t, *J* = 7 Hz, 3H, H-33), 1.19–1.27 (ma, 20H, H-23-32), 1.49 (q, *J* = 7 Hz, 2H, H-22), 2.27 (t, *J* = 7 Hz, 2H, H-21), 2.30–2.38 (m, 1H, H-7α), 2.61–2.68 (dd, *J* = 16, 5 Hz, 1H, H-7β), 3.78–3.83 (m, 1H, H-8), 4.89 (d, *J* = 5 Hz, 1H, H-9), 5.68 (d, *J* = 2 Hz, 1H, H-2), 5.87 (d, *J* = 2 Hz, 1H, H-6), 6.51–6.73 (ma, 3H, H-11, H-14, H-15), 8.84 (s, 1H, H-16), 8.87 (s, 1H, H-17), 8.98 (s, 1H, H-19), 9.22 (s, 1H, H-18). ^13^C-NMR (100 MHz, MeOD): δ (ppm) = 14.5 (C-33), 23.7 (C-32), 26.0 (C-7), 28.5 (C-31), 30.1 (C-30), 30.3 (C-29), 30.4 (C-28), 30.5 (C-27), 30.6 (C-26), 30.7 (C-24-25), 30.8 (C-23), 33.0 (C-22), 34.8 (C-21), 68.7 (C-8), 82.7 (C-9), 95.4 (C-2), 96.2 (C-6), 100.7 (C-4), 115.2 (C-15), 116.0 (C-14), 120.0 (C-11), 132.1 (C-10), 146.1 (C-13), 146.2 (C-12), 156.8 (C-1), 157.5 (C-3), 157.7 (C-5), 176.2 (C-20). IR: ν (cm^−1^) = 2998–3678 (-OH), 1743 (C=O ester).


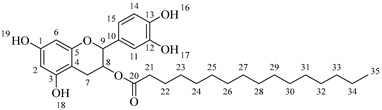


*2-(3,4-Dihydroxyphenyl)-5,7-dihydroxychroman-3-yl hexadecanoate* (**6d**) [36]. Whitish solid. Yield: 77% (0.264 g). m.p. 150 °C. Anal. Calculated for C_31_H_44_O_7_: C, 70.43; H, 8.39; Found: C, 70.47; H, 8.47. ^1^H-NMR (400 MHz, DMSO-*d*_6_): δ (ppm) = 0.84 (t, *J* = 7 Hz, 3H, H-35), 1.19–1.28 (ma, 24H, H-23-34), 1.49 (q, *J* = 7 Hz, 2H, H-22), 2.28 (t, *J* = 7 Hz, 2H, H-21), 2.30–2.39 (m, 1H, H-7α), 2.60–2.67 (dd, *J* = 16, 5 Hz, 1H, H-7β), 3.77–3.83 (m, 1H, H-8), 4.89 (d, *J* = 5 Hz, 1H, H-9), 5.68 (d, *J* = 2 Hz, 1H, H-2), 5.87 (d, *J* = 2 Hz, 1H, H-6), 6.51–6.73 (ma, 3H, H-11, H-14, H-15), 8.84 (s, 1H, H-16), 8.86 (s, 1H, H-17), 8.98 (s, 1H, H-19), 9.21 (s, 1H, H-18). ^13^C-NMR (100 MHz, MeOD): δ (ppm) = 14.4 (C-35), 23.7 (C-34), 26.0 (C-7), 30.1 (C-33), 30.3 (C-32), 30.4 (C-31), 30.5 (C-30), 30.6 (C-29), 30.7 (C-28), 30.8 (C24-27), 30.9 (C-23), 33.0 (C-22), 34.8 (C-21), 61.5 (C-8), 82.7 (C-9), 95.4 (C-2), 96.2 (C-6), 100.7 (C-4), 116.0 (C-15), 116.3 (C-14), 120.0 (C-11), 132.1 (C-10), 146.1 (C-13), 146.2 (C-12), 156.8 (C-1), 157.5 (C-3), 157.7 (C-5), 176.0 (C-20). IR: ν (cm^−1^) = 3013–3700 (-OH), 1743 (C=O ester).


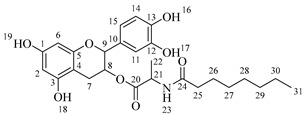


*2-(3,4-Dihydroxyphenyl)-5,7-dihydroxychroman-3-yl octanoylalaninate* (**7a**). Whitish solid. Yield: 81% (0.256 g). m.p. 120 °C. Anal. Calculated for C_26_H_33_NO_8_: C, 64.05; H, 6.82; N, 2.87; Found: C, 64.17; H, 6.80; N, 2.84. ^1^H-NMR (400 MHz, DMSO-*d*_6_): δ (ppm) = 0.94 (t, *J* = 7 Hz, 3H, H-31), 1.31–1.39 (ma, 8H, H-27-30), 1.40 (d, *J* = 7 Hz, 3H, H-22), 1.65 (q, *J* = 7 Hz, 2H, H-26), 2.26 (t, *J* = 7 Hz, 2H, H-25), 2.51–2.59 (m, 1H, H-7α), 2.85–2.94 (dd, *J* = 16, 6 Hz, 1H, H-7β), 3.98–4.05 (m, 1H, H-8), 4.43 (q, *J* = 7 Hz, 1H, H-21), 4.61 (d, *J* = 8 Hz, 1H, H-9), 5.90 (d, *J* = 2 Hz, 1H, H-2), 5.97 (d, *J* = 2 Hz, 1H, H-6), 6.74–6.84 (ma, 3H, H-11, H-14, H-15) 6.88 (d, *J* = 6Hz, 1H, H-23), 8.84 (s, 1H, H-16), 8.86 (s, 1H, H-17), 8.98 (s, 1H, H-19), 9.21 (s, 1H, H-18). ^13^C-NMR (100 MHz, MeOD): δ (ppm) = 14.4 (C-31), 17.3 (C-22), 23.7 (C-30), 26.9 (C-7), 28.5 (C-29), 30.2 (C-28), 30.4 (C-27), 33.0 (C-26), 36.6 (C-25), 52.5 (C-21), 68.8 (C-8), 82.9 (C-9), 94.9 (C-2), 95.5 (C-6), 100.0 (C-4), 115.2 (C-14), 115.8 (C-15), 120.0 (C-11), 131.5 (C-10), 146.2 (C-12-13), 156.9 (C-1), 157.9 (C-3), 170.2 (C-20), 174.9 (C-24). IR: ν (cm^−1^) = 2996–3677 (-OH), 3315 (N-H amide), 1725 (C=O ester), 1642 (C=O amide).


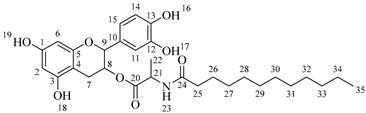


*2-(3,4-Dihydroxyphenyl)-5,7-dihydroxychroman-3-yl dodecanoylalaninate* (**7b**). Whitish solid. Yield: 78% (0.275 g). m.p. 140 °C. Anal. Calculated for C_30_H_41_NO_8_: C, 66.28; H, 7.60; N, 2.58; Found: C, 66.30; H, 7.61; N, 2.54. ^1^H-NMR (400 MHz, DMSO-*d*_6_): δ (ppm) = 0.94 (t, *J* = 7 Hz, 3H, H-35), 1.31–1.39 (ma, 16H, H-27-34), 1.40 (d, *J* = 7Hz, 3H, H-22), 1.65 (q, *J* = 7 Hz, 2H, H-26), 2.26 (t, *J* = 7 Hz, 2H, H-25), 2.51–2.59 (m, 1H, H-7α), 2.85–2.94 (dd, *J* = 16, 6 Hz, 1H, H-7β), 3.98–4.05 (m, 1H, H-8), 4.43 (q, *J* = 7 Hz, 1H, H-21), 4.61 (d, *J* = 8 Hz, 1H, H-9), 5.90 (d, *J* = 2 Hz, 1H, H-2), 5.97 (d, *J* = 2 Hz, 1H, H-6), 6.74–6.84 (ma, 3H, H-11, H-14, H-15) 6.88 (d, *J* = 6Hz, 1H, H-23), 8.84 (s, 1H, H-16), 8.86 (s, 1H, H-17), 8.98 (s, 1H, H-19), 9.21 (s, 1H, H-18). ^13^C-NMR (100 MHz, MeOD): δ (ppm) = 14.4 (C-35), 17.3 (C-22), 23.7 (C-34), 26.9 (C-7), 28.5 (C-33), 30.2 (C-32), 30.4 (C30-31), 30.6 (C-29), 30.7 (C27-28), 33.0 (C-26), 36.6 (C-25), 52.7 (C-21), 68.8 (C-8), 82.9 (C-9), 95.5 (C-2), 96.3 (C-6), 100.8 (C-4), 115.3 (C-14), 116.0 (C-15), 120.0 (C-11), 132.2 (C-10), 146.2 (C-12-13), 156.9 (C-1), 157.9 (C-3), 170.2 (C-20), 175.0 (C-24). IR: ν (cm^−1^) = 3000–3677 (-OH), 3320 (N-H amide), 1725 (C=O ester), 1642 (C=O amide).


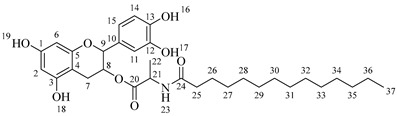


*2-(3,4-Dihydroxyphenyl)-5,7-dihydroxychroman-3-yl tetradecanoylalaninate* (**7c**). Whitish solid. Yield: 82% (0.304 g). m.p. 135 °C. Anal. Calculated for C_32_H_45_NO_8_: C, 67.23; H, 7.93; N, 2.45; Found: C, 67.22; H, 7.96; N, 2.43. ^1^H-NMR (400 MHz, DMSO-*d*_6_): δ (ppm) = 0.94 (t, *J* = 7 Hz, 3H, H-37), 1.30–1.39 (ma, 20H, H-27-36), 1.40 (d, *J* = 7 Hz, 3H, H-22), 1.64 (q, *J* = 7 Hz, 2H, H-26), 2.35 (t, *J* = 7 Hz, 2H, H-25), 2.51–2.59 (m, 1H, H-7α), 2.86–2.94 (dd, *J* = 16, 6 Hz, 1H, H-7β), 3.98–4.05 (m, 1H, H-8), 4.43 (q, *J* = 7 Hz, 1H, H-21), 4.59 (d, *J* = 8 Hz, 1H, H-9), 5.90 (d, *J* = 2 Hz, 1H, H-2), 5.97 (d, *J* = 2 Hz, 1H, H-6), 6.74–6.84 (ma, 3H, H-11, H-14, H-15) 6.88 (d, *J* = 6 Hz, 1H, H-23), 8.84 (s, 1H, H-16), 8.87 (s, 1H, H-17), 8.98 (s, 1H, H-19), 9.22 (s, 1H, H-18). ^13^C-NMR (100 MHz, MeOD): δ (ppm) = 14.4 (C-37), 17.4 (C-22), 23.7 (C-36), 26.0 (C-35), 26.3 (C-34), 26.9 (C-7), 28.5 (C-33), 30.2 (C-32), 30.3 (C30) 30.4 (C-31), 30.5 (C-30), 30.6 (C-29), 30.7 (C27-28), 33.0 (C-26), 36.6 (C-25), 52.0 (C-21), 68.8 (C-8), 82.9 (C-9), 95.5 (C-2), 96.5 (C-6), 100.8 (C-4), 115.3 (C-14), 116.0 (C-15), 120.0 (C-11), 132.2 (C-10), 146.2 (C-12-13), 156.9 (C-1), 157.9 (C-3), 170.2 (C-20), 174.9 (C-24). IR: ν (cm^−1^) = 3998–3694 (-OH), 3320 (N-H amide), 1730 (C=O ester), 1645 (C=O amide).


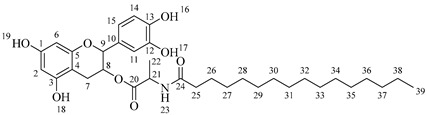


*2-(3,4-Dihydroxyphenyl)-5,7-dihydroxychroman-3-yl hexadecanoylalaninate* (**7d**). Whitish solid. Yield: 79% (0.308 g). m.p. 150 °C. Anal. Calculated for C_34_H_49_NO_8_: C, 68.09; H, 8.24; N, 2.34; Found: C, 68.12; H, 8.27; N, 2.39. ^1^H-NMR (400 MHz, DMSO-*d*_6_): δ (ppm) = 0.94 (t, *J* = 7 Hz, 3H, H-39), 1.27–1.39 (ma, 24H, H-27-38), 1.40 (d, *J* = 7 Hz, 3H, H-22), 1.65 (q, *J* = 7 Hz, 2H, H-26), 2.26 (t, *J* = 7 Hz, 2H, H-25), 2.32–2.43 (m, 1H, H-7α), 2.86–2.94 (dd, *J* = 16, 6 Hz, 1H, H-7β), 3.98–4.05 (m, 1H, H-8), 4.43 (q, *J* = 7 Hz, 1H, H-21), 4.59 (d, *J* = 8 Hz, 1H, H-9), 5.91 (d, *J* = 2 Hz, 1H, H-2), 5.98 (d, *J* = 2 Hz, 1H, H-6), 6.74–6.84 (ma, 3H, H-11, H-14, H-15) 6.90 (d, *J* = 6Hz, 1H, H-23), 8.84 (s, 1H, H-16), 8.87 (s, 1H, H-17), 8.98 (s, 1H, H-19), 9.22 (s, 1H, H-18). ^13^C-NMR (100 MHz, MeOD): δ (ppm) = 14.4 (C-39), 17.3 (C-22), 23.7 (C-38), 26.9 (C-7), 30.2 (C-37), 30.3 (C-36), 30.4 (C-35), 30.6 (C-34), 30.8 (C-33), 30.8 (C-27-32), 33.1 (C-26), 36.6 (C-25), 52.8 (C-21), 68.8 (C-8), 82.9 (C-9), 95.5 (C-2), 96.5 (C-6), 100.8 (C-4), 115.3 (C-14), 116.0 (C-15), 120.0 (C-11), 132.2 (C-10), 146.2 (C-12-13), 156.9 (C-1), 157.9 (C-3), 170.3 (C-20), 175.0 (C-24). IR: ν (cm^−1^) = 3998–3700 (-OH), 3315 (N-H amide), 1730 (C=O ester), 1645 (C=O amide).

### 3.3. Water Solubility

Water solubility was obtained by saturating water with the final compounds **6a**–**6d** and **7a**–**7d**, then taking a precise volume, lyophilizing and weighing in order to determine the exact mass of the product solubilized in the exact volume of water taken. Solubility in water was expressed in mol/L (M).

### 3.4. Surfactant Properties

Surface tension (γ) was obtained by the Wilhelmy plate method, using a Force Tensiometer KRUSS at 25 °C. For the bi-modular and tri-modular catechin derivatives, compounds **6a**–**6d** and **7a**–**7d**, solutions at different concentrations were prepared, and the surface tension was measured at each concentration. The curve log C vs. γ allowed us to calculate critical aggregate concentration (CAC), minimum occupied area per molecule at the air–water interface (σ) and the surface tension (γ_w_) that remains constant after the CAC was obtained. Each measurement was conducted in triplicate.

### 3.5. Radical Scavenging Activity

The radical scavenging activity of the anhydrous catechin and the hemi-synthesis compounds **6a**–**6d** and **7a**–**7d** was measured at λ = 415 nm by UV-visible spectroscopy according to the DPPH (2,2-diphenyl 1-picryldhydrazyl) method using a PERKIN ELMER UV/VIS Lambda 365 Spectrophotometer. This method measures the antioxidant capacity of a compound to reduce the purple-colored DPPH radical solution to yellow-colored DPPH-H. DPPH solution was prepared at a concentration of 10^−4^ M in methanol. The IC_50_ was calculated using the formula IC_50_ = (y − b)/a obtained from the equation of the line y = ax + b where a is the directing coefficient and b the y-intercept. Each measurement was conducted in triplicate.

### 3.6. Antibacterial Activity

The antibacterial activity of the anhydrous catechin and bi-modular and tri-modular catechin derivatives **6a**–**6d** and **7a**–**7d** was evaluated by a resazurin reduction test. Resazurin is a blue colorant that reduces to the fluorescent pink resorufin when metabolized by the cells from bacterial respiratory chains. The metabolic activity of the cells was measured by reading the fluorescence over 72 h using a BioTek FLx800 Fluorescence Microplate Reader.

Strains of *Escherichia coli* were cultured for 12 h on solid LB medium (Luria–Bertani) at 35 °C. The next day, some colonies were taken and placed in a nutrient broth and incubated at 35 °C until an optical density of 0.4 was obtained. The tests were performed in a 96-well microplate in which different solutions of catechin or compounds **6a**–**6d** and **7a**–**7d** in DMSO, the inoculum, and the resazurin were introduced. A control with ethoxyethanol was performed. A positive control to verify the growth of the bacteria in the absence of catechin or hemi-synthesis compound was performed as well. The IC_50_ was calculated using the formula IC_50_ = (y − b)/a obtained from the equation of the line y = ax + b, where a is the directing coefficient and b the y-intercept. Each measurement was conducted in triplicate.

## Figures and Tables

**Table 1:** Water solubility of anhydrous catechin and the bi-modular and tri-modular catechin derivatives **6a**–**6d** and **7a**–**7d**.

Water Solubility (M)								
**Catechin [23]**	**6a**	**6b**	**6c**	**6d**	**7a**	**7b**	**7c**	**7d**
15.7 × 10^−3^	6.0 × 10^−3^	4.8 × 10^−3^	4.8 × 10^−3^	2.4 × 10^−4^	1.3 × 10^−3^	3.6 × 10^−4^	5.8 × 10^−4^	2.2 × 10^−5^

**Table 2:** Critical aggregation concentration (CAC), surface tension from CAC (γ_w_), and minimum occupied area per molecule at the air–water interface (σ) for compounds **6a**–**6d** and **7a**–**7d**.

Surfactant Properties								
	**6a**	**6b**	**6c**	**6d**	**7a**	**7b**	**7c**	**7d**
**CAC (M)**	8.9 × 10^−4^	6.8 × 10^−4^	5.9 × 10^−4^	1 × 10^−5^	1 × 10^−4^	0.5 × 10^−4^	0.4 × 10^−4^	2 × 10^−6^
**γ_w_ (mN/m)**	43	49	41	42	35	32	33	32
**σ (Å^2^/molecule)**	14	17	23	29	8	10	19	45

**Table 3:** Radical scavenging activity expressed as IC_50_ values of anhydrous catechin, compounds **6a**–**6d** and **7a**–**7d**, and other natural antioxidant polyphenols present in wood (**a**–**g**) and synthetic antioxidant compounds presenting a phenol group (**h**–**i**).

Radical Scavenging Activity: IC_50_ (μg/mL)	
**catechin**	2.43 ± 0.03
**6a**	2.11 ± 0.02
**6b**	2.27 ± 0.05
**6c**	2.10 ± 0.07
**6d**	3.28 ± 0.06
**7a**	2.06 ± 0.04
**7b**	2.42 ± 0.06
**7c**	2.01 ± 0.05
**7d**	26.73 ± 0.43
**a. tannic acid** [24]	23.65
**b. caffeic acid** [24]	10.64
**c. pinosylvin** [25]	0.17 ± 0.21
**d. secoisolariciresinol** [26]	9.39 ± 0.01
**e. taxifolin** [26]	10.06 ± 1.08
**f. quercetin** [26]	1.59 ± 0.02
**g. gallic acid** [26]	2.04 ± 0.03
**h. butylated hydroxyanisole (BHA)** [27]	21.3 ± 0.12
**i. butylated hydroxytoluene (BHT)** [27]	12.8 ± 0.08

**Table 4:** Antibacterial activity against *Escherichia coli* expressed as IC_50_ values for anhydrous catechin and bi-modular and tri-modular catechin derivatives **6a**–**6d** and **7a**–**7d**.

Antibacterial Activity Against *E. coli*: IC_50_ (mM)								
**Catechin**	**6a**	**6b**	**6c**	**6d**	**7a**	**7b**	**7c**	**7d**
36 ± 2	26 ± 3	26 ± 4	25 ± 1	55 ± 1	32 ± 3	34 ± 3	23 ± 1	232 ± 4

## Data Availability

The original contributions presented in this study are included in the article/Supplementary Material. Further inquiries can be directed to the corresponding author.

## Supplementary Materials

The following supporting information can be downloaded at: https://www.mdpi.com/article/10.3390/molecules30214223/s1, Figure S1. ^1^H-NMR, ^13^C-NMR and FTIR spectra of *2-(3,4-dihydroxyphenyl)-5,7-dihydroxychroman-3-yl octanoylalaninate* (**7a**); Figure S2. ^1^H-NMR, ^13^C-NMR and FTIR spectra of *2-(3,4-dihydroxyphenyl)-5,7-dihydroxychroman-3-yl dodecanoylalaninate* (**7b**); Figure S3. ^1^H-NMR, ^13^C-NMR and FTIR spectra of *2-(3,4-dihydroxyphenyl)-5,7-dihydroxychroman-3-yl tetradecanoylalaninate* (**7c**); Figure S4. ^1^H-NMR, ^13^C-NMR and FTIR spectra of *2-(3,4-dihydroxyphenyl)-5,7-dihydroxychroman-3-yl hexadecanoylalaninate* (**7d**).

## Abbreviations

The following abbreviations are used in this manuscript:

AcCl	Acetyl chloride
BHA	Butylated hydroxyanisole
BHT	Butylated hydroxytoluene
CAC	Critical aggregation concentration
CH_2_Cl_2_	Dichloromethane
DPPH	2,2-diphenyl-1-picrylhydrazyl
Et_3_N	Triethylamine
FTIR	Fourier transform infrared spectroscopy
H_2_O	Water
H_2_SO_4_	Sulfuric acid
LB	Luria–Bertani
MeOH	Methanol
Me-THF	Methyl-tetrahydrofuran
MW	Microwave
NaCl	Sodium chloride
NaHCO_3_	Sodium bicarbonate
NaOH	Sodium hydroxide
NMR	Nuclear magnetic resonance
THF	Tetrahydrofuran
TLC	Thin layer chromatography

## References

[1] El-Saadony M.T., Yang T., Saad A.M., Alkafaas S.S., Elkafas S.S., Eldeeb G.S., Mohammed D.M., Salem H.M., Korma S.A., Loutfy S.A. (2024). Polyphenols: Chemistry, Bioavailability, Bioactivity, Nutritional Aspects and Human Health Benefits: A Review. Int. J. Biol. Macromol..

[2] Bae J., Kim N., Shin Y., Kim S.-Y., Kim Y.-J. (2020). Activity of Catechins and Their Applications. Biomed. Dermatol..

[3] Pedro A.C., Maciel G.M., Rampazzo Ribeiro V., Haminiuk C.W.I. (2020). Fundamental and Applied Aspects of Catechins from Different Sources: A Review. Int. J. Food Sci. Technol..

[4] Coppo E., Marchese A. (2014). Antibacterial Activity of Polyphenols. Curr. Pharm. Biotechnol..

[5] Scicutella F., Mannelli F., Daghio M., Viti C., Buccioni A. (2021). Polyphenols and Organic Acids as Alternatives to Antimicrobials in Poultry Rearing: A Review. Antibiotics.

[6] Chen M., Zhao Z., Meng H., Yu S. (2017). The Antibiotic Activity and Mechanisms of Sugar Beet (Beta Vulgaris) Molasses Polyphenols against Selected Food-Borne Pathogens. LWT.

[7] Dai W., Ruan C., Zhang Y., Wang J., Han J., Shao Z., Sun Y., Liang J. (2020). Bioavailability Enhancement of EGCG by Structural Modification and Nano-Delivery: A Review. J. Funct. Foods.

[8] Rashidinejad A., Boostani S., Babazadeh A., Rehman A., Rezaei A., Akbari-Alavijeh S., Shaddel R., Jafari S.M. (2021). Opportunities and Challenges for the Nanodelivery of Green Tea Catechins in Functional Foods. Food Res. Int..

[9] Sasaki G.Y., Vodovotz Y., Yu Z., Bruno R.S. (2022). Catechin Bioavailability Following Consumption of a Green Tea Extract Confection Is Reduced in Obese Persons without Affecting Gut Microbial-Derived Valerolactones. Antioxidants.

[10] De Souza J.E., Casanova L.M., Costa S.S. (2015). Bioavailability of Phenolic Compounds: A Major Challenge for Drug Development?. Rev. Fitos.

[11] Liu J., Lu J., Wen X., Kan J., Jin C. (2015). Antioxidant and Protective Effect of Inulin and Catechin Grafted Inulin against CCl4-Induced Liver Injury. Int. J. Biol. Macromol..

[12] Oliver S., Jofri A., Thomas D.S., Vittorio O., Kavallaris M., Boyer C. (2017). Tuneable Catechin Functionalisation of Carbohydrate Polymers. Carbohydr. Polym..

[13] Li D., Wang R., Ma Y., Yu D. (2023). Covalent Modification of (+)-Catechin to Improve the Physicochemical, Rheological, and Oxidative Stability Properties of Rice Bran Protein Emulsion. Int. J. Biol. Macromol..

[14] Abdjan M.I., Rosyda M., Aminah N.S., Kristanti A.N., Siswanto I., Shehzad W., Siddiqui H., Wardana A.P., Indriani, Saputra M.A. (2024). Synthesis of Ester Derivatives of Catechin Isolated from Uncaria Gambir and Their Anticancer Activity. Eng. Sci..

[15] Zhou J., Tian Q., Ma Y., Wang Y., Huo Q. (2021). Lipase-Catalyzed Selective Esterification of Catechin. Materials Express.

[16] McOmie J.F.W. (1976). Protective Groups in Organic Chemistry.

[17] Wuts P.G.M. (2014). Greene’s Protective Groups in Organic Synthesis.

[18] Muskifa J., Khan R.A., Khan I.A., Ferreira D. (2004). Benzylation of Flavan-3-Ols (Catechins). Org. Prep. Proced. Int..

[19] Nakamura S., Oyama K.-I., Kondo T., Yoshida K. (2007). ANALYSIS OF BENZYLATION PRODUCTS OF (+)-CATECHIN. Heterocycles.

[20] Hergert H.L., Kurth E.F. (1953). The isolation and properties of catechol from white fir bark. J. Org. Chem..

[21] Raab T., Barron D., Arce Vera F., Crespy V., Oliveira M., Williamson G. (2010). Catechin Glucosides: Occurrence, Synthesis, and Stability. J. Agric. Food Chem..

[22] Yeom C.E., Lee S.Y., Kim Y.J., Kim B.M. (2005). Mild and Chemoselective Deacetylation Method Using a Catalytic Amount of Acetyl Chloride in Methanol. Synlett.

[23] Cuevas-Valenzuela J., González-Rojas Á., Wisniak J., Apelblat A., Pérez-Correa J.R. (2015). Solubility of (+)-Catechin in Water and Water-Ethanol Mixtures within the Temperature Range 277.6-331.2K: Fundamental Data to Design Polyphenol Extraction Processes. Fluid Phase Equilib..

[24] Gulcin İ., Alwasel S.H. (2023). DPPH Radical Scavenging Assay. Processes.

[25] Sinyeue C., Maerker L., Guentas L., Medevielle V., Bregier F., Chaleix V., Sol V., Lebouvier N. (2023). Polyphenol Content, Antioxidant, and Antibiotic Activities of Pinus Caribaea Morelet Forestry Coproducts. Nat. Prod. Commun..

[26] Gérardin P., Hentges D., Gérardin P., Vinchelin P., Dumarçay S., Audoin C., Gérardin-Charbonnier C. (2023). Knotwood and Branchwood Polyphenolic Extractives of Silver Fir, Spruce and Douglas Fir and Their Antioxidant, Antifungal and Antibacterial Properties. Molecules.

[27] Djouahri A., Sebiane S., Kellou F., Lamari L., Sabaou N., Baaliouamer A., Boudarene L. (2017). Inhibitory Effect on Corrosion of Carbon Steel in Acidic Media, Antioxidant, Antimicrobial, Anti-5-Lipooxygenase and Anti-Xanthine Oxidase Activities of Essential Oil from Tetraclinis Articulata (Vahl) Masters Leaves. J. Essent. Oil Res..

[28] Abuga I., Sulaiman S.F., Abdul Wahab R., Ooi K.L., Abdull Rasad M.S.B. (2020). In Vitro Antibacterial Effect of the Leaf Extract of Murraya Koenigii on Cell Membrane Destruction against Pathogenic Bacteria and Phenolic Compounds Identification. Eur. J. Integr. Med..

[29] Lobiuc A., Pavăl N.E., Mangalagiu I.I., Gheorghiță R., Teliban G.C., Amăriucăi-Mantu D., Stoleru V. (2023). Future Antimicrobials: Natural and Functionalized Phenolics. Molecules.

[30] Markossian S., Grossman A., Baskir H. (2021). Biosensor Assays for Measuring the Kinetics of G-Protein and Arrestin-Mediated Signaling in Live Cells. Assay Guidance Manual.

[31] Biondo C. (2023). Bacterial Antibiotic Resistance: The Most Critical Pathogens. Pathogens.

[32] Breijyeh Z., Jubeh B., Karaman R. (2020). Resistance of Gram-Negative Bacteria to Current Antibacterial Agents and Approaches to Resolve It. Molecules.

[33] Renzetti A., Betts J.W., Fukumoto K., Rutherford R.N. (2020). Antibacterial Green Tea Catechins from a Molecular Perspective: Mechanisms of Action and Structure-Activity Relationships. Food Funct..

[34] Turcotte P., Saheb S.A. (1978). Activité Antimicrobienne d’antioxydants Phénoliques. Can. J. Microbiol..

[35] Arellano H., Nardello-Rataj V., Szunerits S., Boukherroub R., Fameau A.L. (2023). Saturated Long Chain Fatty Acids as Possible Natural Alternative Antibacterial Agents: Opportunities and Challenges. Adv. Colloid Interface Sci..

[36] Uesato S., Taniuchi K., Kumagai A., Nagaoka Y., Seto R., Hara Y., Tokuda H., Nishino H. (2003). Inhibitory Effects of 3-O-Acyl-(+)-Catechins on Epstein-Barr Virus Activation. Chem. Pharm. Bull..

