# Supplementary material for: Rapid Hemi-Synthesis of Multifunctional Amphiphilic Derivatives Based on Polyphenolic Extractives: Surface Activity, Antioxidant and Antibacterial Properties
Source: Molecules. 2025 Oct 29;30(21):4223. doi: 10.3390/molecules30214223 (PMC12608305; doi:10.3390/molecules30214223)
Supplement: Supplementary file 1 [file molecules-30-04223-s001.zip › molecules-3912673-supplementary.pdf]

molecules

MDPI

Supplementary Material

Totally green hemi-synthesis of multifunctional amphiphilic derivatives based on polyphenolic extractives: surface activity, antioxidant and antibacterial properties

Maria Celeste Ruiz [1], Pauline Gérardin[1], Georges Eid[1], Jean-Luc Blin3, Catherine Humeau-Virot[2] and Christine Gérardin-Charbonnier [1,*]

[1] Université de Lorraine, INRAE, Laboratoire d'Etudes et de Recherche sur le Matériau Bois (LERMAB), Faculté des Sciences et Technologies, BP 70239, 54506 Vandœuvre-lès-Nancy, France
[2] Université de Lorraine, Laboratoire Réactions et Génie des Procédés (LRGP), Université de Lorraine,2 avenue de la Forêt de Haye, TSA40602, F-54518 Vandœuvre-lès-Nancy, France, BP 70239, France
[3] Université de Lorraine, Laboratoire Lorrain de Chimie Moléculaire UMR CNRS 7053 L2CM, 54500 Vandoeuvre-lès-Nancy, France
* Correspondence: christine.gerardin@univ-lorraine.fr; Tel.: + 33 (0)3 72 74 52 34

List of Figures:

Figure S1. ^{1}H-NMR, ^{13}C-NMR and FTIR spectra of *2-(3,4-dihydroxyphenyl)-5,7-dihydroxychroman-3-yl octanoylalaninate* (**7a**).

Figure S2. ^{1}H-NMR, ^{13}C-NMR and FTIR spectra of *2-(3,4-dihydroxyphenyl)-5,7-dihydroxychroman-3-yl dodecanoylalaninate* (**7b**).

Figure S3. ^{1}H-NMR, ^{13}C-NMR and FTIR spectra of *2-(3,4-dihydroxyphenyl)-5,7-dihydroxychroman-3-yl tetradecanoylalaninate* (**7c**).

Figure S4. ^{1}H-NMR, ^{13}C-NMR and FTIR spectra of *2-(3,4-dihydroxyphenyl)-5,7-dihydroxychroman-3-yl hexadecanoylalaninate* (**7d**).

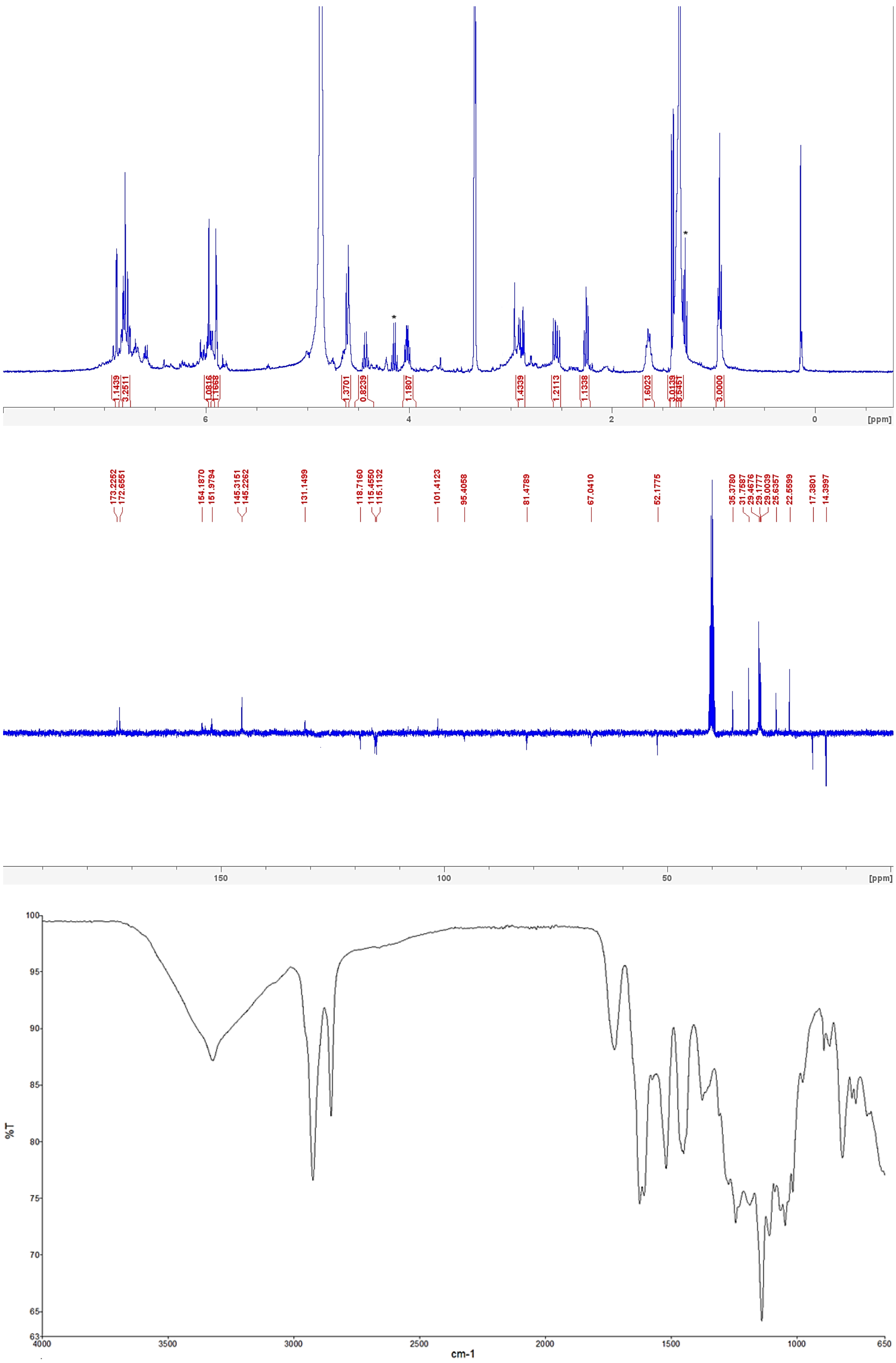

Figure S1. ^{1}H-NMR (MeOD), ^{13}C-NMR (DMSO-d$_6$) and FTIR spectra of *2-(3,4-dihydroxyphenyl)-5,7-dihydroxychroman-3-yl octanoylalaninate* (**7a**).

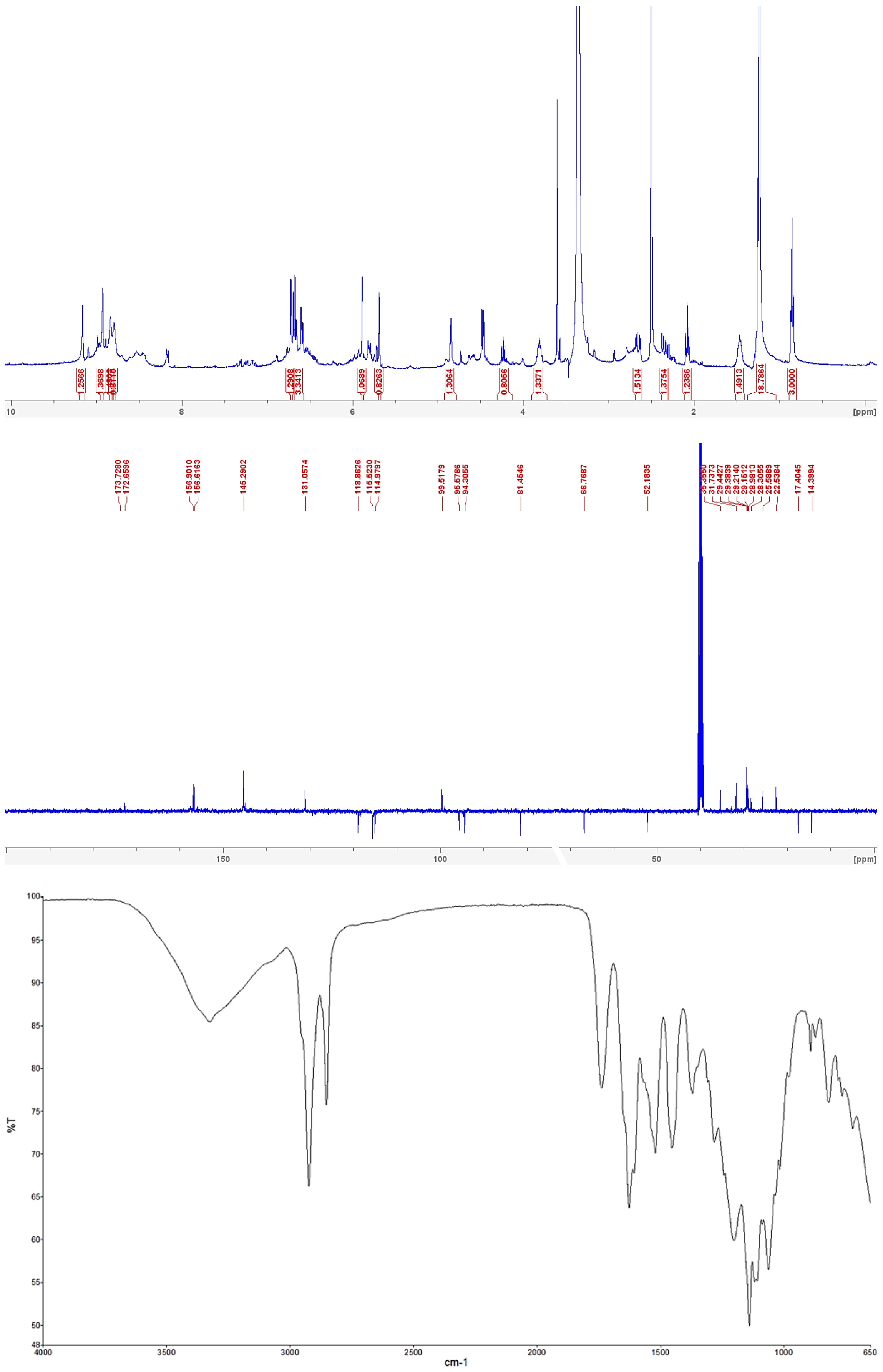

Figure S2. ^{1}H-NMR (DMSO-d6), ^{13}C-NMR (DMSO-d6) and FTIR spectra of *2-(3,4-dihydroxyphenyl)-5,7-dihydroxychroman-3-yl dodecanoylalaninate* (**7b**).

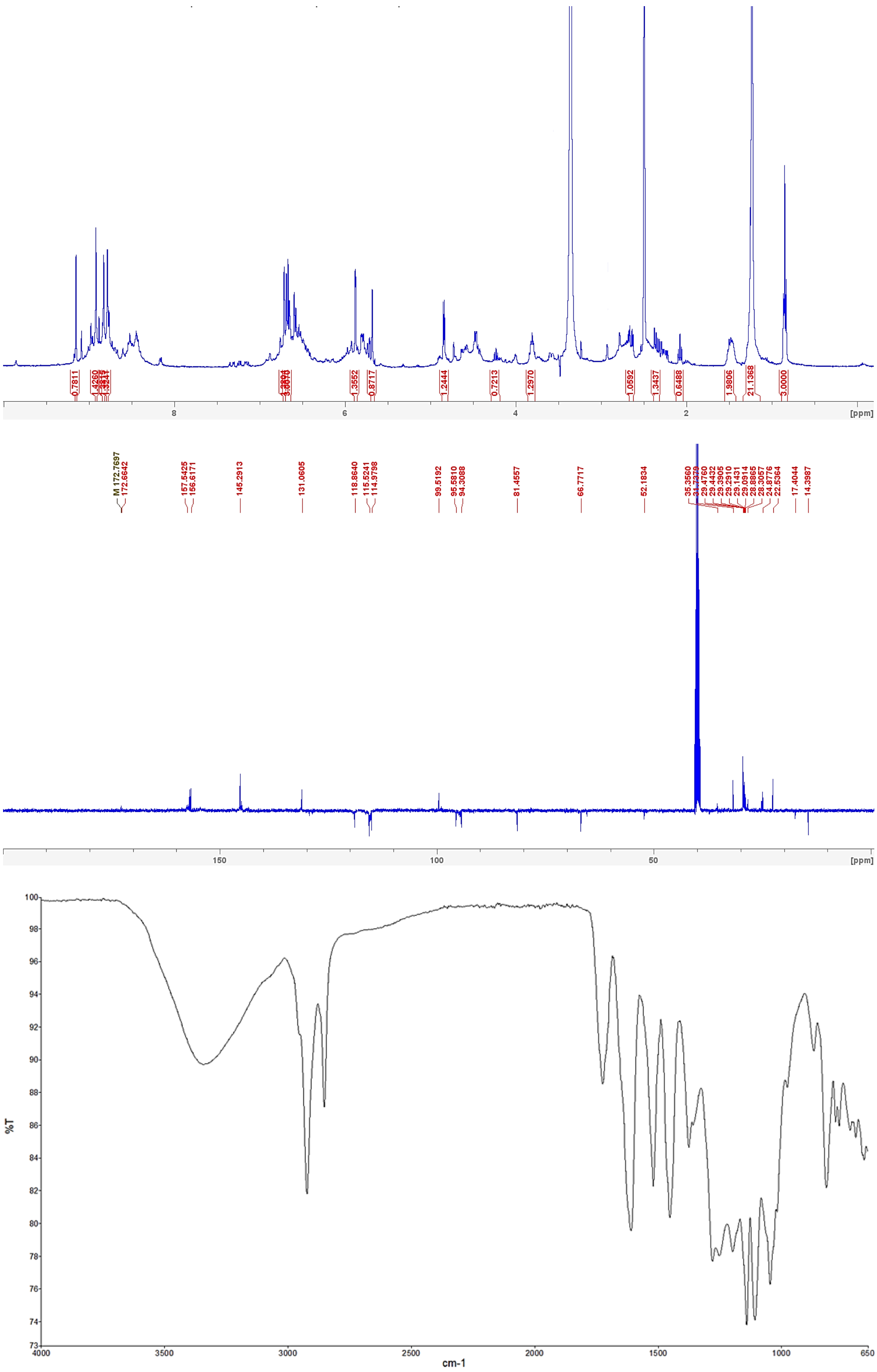

Figure S3. ^{1}H-NMR (DMSO-d$_6$), ^{13}C-NMR (DMSO-d$_6$) and FTIR spectra of *2-(3,4-dihydroxyphenyl)-5,7-dihydroxychroman-3-yl tetradecanoylalaninate* (**7c**).

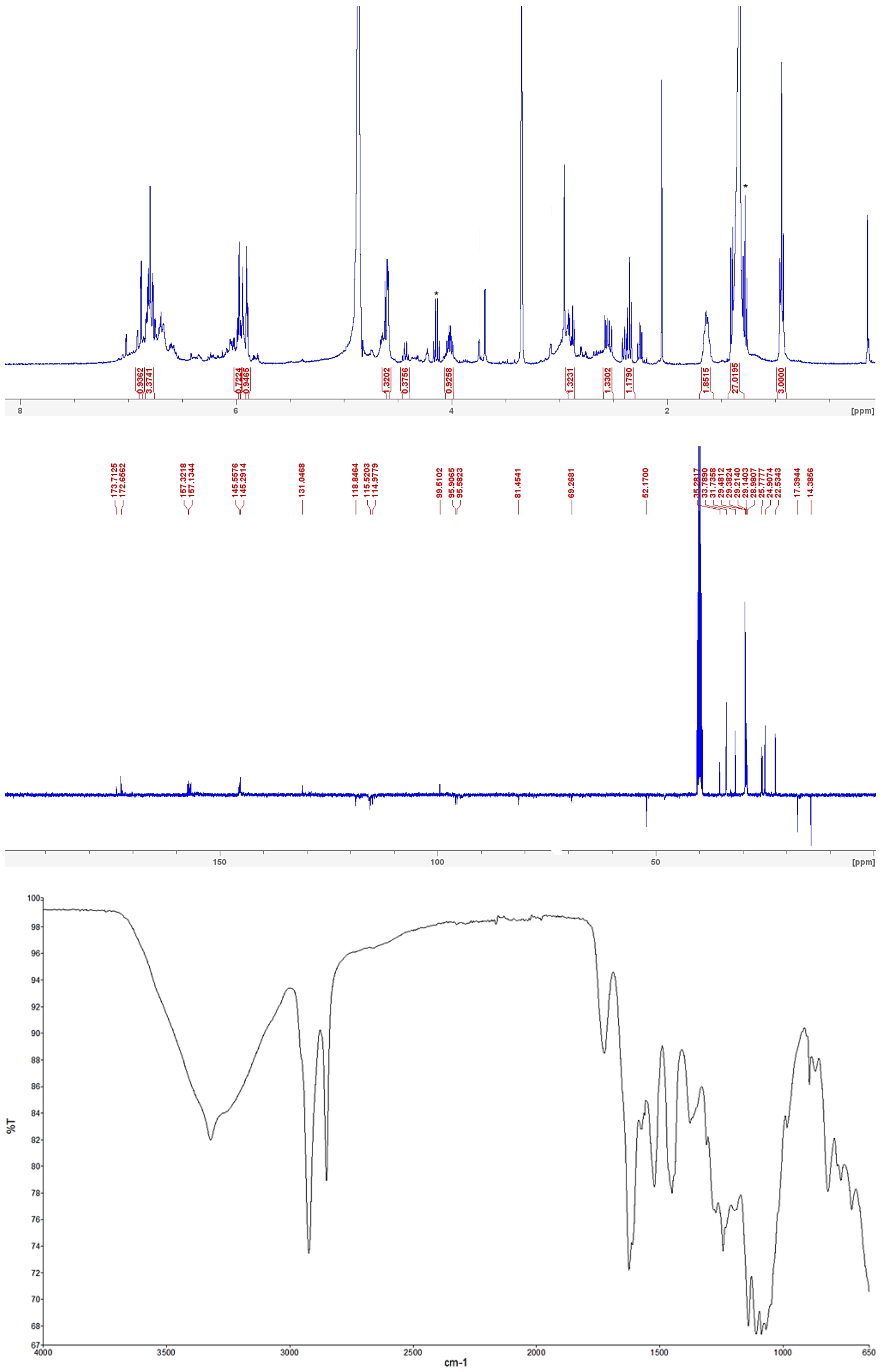

Figure S4. ^{1}H-NMR (MeOD), ^{13}C-NMR (DMSO-d_6) and FTIR spectra of *2-(3,4-dihydroxyphenyl)-5,7-dihydroxychroman-3-yl hexadecanoylalaninate* (**7d**).
